# Ambient Air Pollution and Congenital Heart Disease: Updated Evidence and Future Challenges

**DOI:** 10.3390/antiox14010048

**Published:** 2025-01-03

**Authors:** Francesca Gorini, Alessandro Tonacci

**Affiliations:** Institute of Clinical Physiology, National Research Council, 56124 Pisa, Italy; alessandro.tonacci@cnr.it

**Keywords:** congenital heart disease, maternal exposure, ambient air pollution, carbon monoxide, nitrogen dioxide, ozone, particulate matter, sulfur dioxide, Artificial Intelligence, Internet of Things

## Abstract

Congenital heart disease (CHD) represents the major cause of infant mortality related to congenital anomalies globally. The etiology of CHD is mostly multifactorial, with environmental determinants, including maternal exposure to ambient air pollutants, assumed to contribute to CHD development. While particulate matter (PM) is responsible for millions of premature deaths every year, overall ambient air pollutants (PM, nitrogen and sulfur dioxide, ozone, and carbon monoxide) are known to increase the risk of adverse pregnancy outcomes. In this literature review, we provide an overview regarding the updated evidence related to the association between maternal exposure to outdoor air pollutants and CHD occurrence, also exploring the underlying biological mechanisms from human and experimental studies. With the exception of PM, for which there is currently moderate evidence of its positive association with overall CHD risk following exposure during the periconception and throughout pregnancy, and for ozone which shows a signal of association with increased risk of pooled CHD and certain CHD subtypes in the periconceptional period, for the other pollutants, the data are inconsistent, and no conclusion can be drawn about their role in CHD onset. Future epidemiological cohort studies in countries with different degree of air pollution and experimental research on animal models are warranted to gain a comprehensive picture of the possible involvement of ambient air pollutants in CHD etiopathogenesis. While on the one hand this information could also be useful for timely intervention to reduce the risk of CHD, on the other hand, it is mandatory to scale up the use of technologies for pollutant monitoring, as well as the use of Artificial Intelligence for data analysis to identify the non-linear relationships that will eventually exist between environmental and clinical variables.

## 1. Introduction

Congenital heart disease (CHD), defined as a group of defects in the structure of the heart and great vessels, is the most common malformation at birth, with 13.3 million affected patients in 2019 [1,2,3,4]. A recent estimate indicated a global prevalence for CHD of 9.4 cases per 1000 newborns in the period 2010–2017, with a significant increase compared to the years 1970–1974 (4.5 cases per 1000) [4]. Mild lesions (atrial septal defects—ASD, ventricular septal defects—VSD, and patent ductus arteriosus—PDA) account for 93.4% of the increased overall prevalence, probably as a consequence of an improved prenatal detection rate via fetal echocardiogram, especially in high-income countries [4,5]. On the other hand, regional differences in CHD prevalence have been identified, Africa being characterized by the lowest CHD prevalence and Asia by the highest [4], further highlighting the role for pre- and perinatal screening in relation to these estimates. Despite advances in early diagnosis and congenital heart surgery over the past three decades, which have led to a significant decline in the global crude mortality rate for CHD (from 7.1 per 100,000 in 1990 to 2.8 per 100,000 in 2019, with a 60.4% decrease overall), CHD still represents the leading cause of infant mortality due to congenital abnormalities worldwide [3,6,7]. In particular, in the period 1990–2019, the number of fatalities attributable to CHD decreased in world regions, except for low-sociodemographic index regions, where an increase of 23.7% was recorded [3].

If genetics (chromosomal abnormalities, copy number variations, and single gene disorders) plays a central role in CHD pathogenesis, contributing to approximately 40% of CHD cases, and exposure to environmental determinants may account for an estimated 5%, the remaining 55% of cases have an unknown etiology, possibly being of multifactorial origin [8,9]. Among environmental/non-genetic factors, some of them are considered to be non-modifiable (maternal rheumatologic and metabolic disorders, maternal infections, and medication use in pregnancy), while modifiable risk factors include features attributable to mothers, including dietary deficiency (i.e., low intake of vitamin B_12_, vitamin B_2_, niacin, and iron [10]), overweight/obesity [11], smoking and alcohol consumption [12,13], and exposure to environmental contaminants, i.e., toxic metals and air pollutants [7,14,15,16].

Ambient air pollutants, which include particulate matter (PM), nitrogen dioxide (NO_2_), sulfur dioxide (SO_2_), carbon monoxide (CO), and ozone (O_3_), collectively represent the second highest risk determinant for noncommunicable diseases [17,18]. Outdoor air pollution has been estimated to be responsible for 4.2 million premature deaths worldwide in 2019, 68% of which are related to ischemic heart disease and stroke, and with the majority (89%) occurring in low- and middle-income world regions [17]. Furthermore, maternal exposure to ambient air pollutants during pregnancy appears to contribute to a higher risk of adverse birth outcomes, such as preterm birth (PTB), low birth weight (LBW), and small per gestational age (SGA), and with certain congenital anomalies, i.e., limb and orofacial defects [19,20,21]. Accumulating evidence also suggests the possibility of an association between maternal exposure to ambient air pollution, particularly during the first trimester of pregnancy, which is considered to be a critical window for fetal cardiac development, and the onset of CHD, although the results are somewhat inconsistent [15,22]. Air pollutants could be involved in CHD development through diverse mechanisms including oxidative stress, inflammation, epigenetic modifications, and changes in gene expression [22]. Given the relevant threat of air pollutants to human health, especially during childhood, and the clinical and socioeconomic impact of CHD, this comprehensive literature review aims at discussing the current knowledge on the risk of CHD associated with prenatal exposure to ambient air pollution and the plausibility of biological processes potentially involved. We also explore the prospects for reducing the prevalence of CHD and the resulting healthcare costs through new strategies for monitoring air quality and analyzing related data based on new technologies and recent methodologies.

## 2. The Relationship Between Ambient Air Pollution and Congenital Heart Disease

In recent years, air pollution has increasingly become a global public health concern due to various factors including a rapidly growing population, urbanization, industrialization, and the lack of effective measures to control atmospheric pollutant emissions [21]. It is estimated that, in 2019, almost all of the world’s population (99%) was exposed to levels of ambient air pollution above the air quality guidelines set by the World Health Organization (WHO) to ensure an adequate response to the continuing threat of air pollution to public health [23,24]. In 2020, exposure to concentrations of fine particulate matter (particles with an aerodynamic diameter less than 2.5 µM—PM_2.5_) exceeding the 2021 WHO guideline level caused 238,000 premature deaths in the 27 Member States of the European Union [24,25].

Air pollutants, classified into outdoor or indoor contaminants, are defined as any chemical, physical, or biological agent able to modify the natural atmospheric environment [26]. Multiple sources, mainly anthropogenic, are responsible for the generation of air pollution, i.e., motor vehicles, manufacturing and extractive industries, agriculture, power plants, forest fires, and volcanoes [25,26]. Once airborne, pollutants may react with other chemicals, producing other pollutants, and are subject to dispersion, depending on meteorological conditions, source, and local and regional geographical features, as well as deposition processes (precipitation, scavenging, and sedimentation), which in turn lead to the accumulation of air pollutants in other environmental matrices [27,28]. The effects of outdoor pollution on humans depend on various variables including the composition and concentration of pollutants, duration of exposure, and individual susceptibility [29]. Therefore, common air quality assessments based on high-end static sensor stations, while able to produce aggregated information about air pollutants, do not provide data on air pollution exposure at the individual level [29]. On the other hand, low-cost portable sensors allow us to classify individual exposure into outdoor and indoor settings, providing personalized information on exposure to air pollutants in daily commutes, validating and even integrating the data with measurements from high-end reference monitoring stations [29,30].

Based on the so-called “Barker-hypothesis”, also known as the “fetal origin of adult disease”, each environmental determinant, including pollutants, able to interfere with the physiological expression of fetal genes during intrauterine development may lead to alterations in fetal structure and function and may also increase susceptibility to certain chronic conditions, including metabolic syndrome and associated cardiovascular complications (e.g., coronary heart disease, and stroke) during adulthood [31,32]. Adverse pregnancy outcomes, such as LBW, are considered a proxy measure of a compromised intrauterine environment and have been linked to a range of cardiovascular and metabolic disorders in adult life [33]. The placenta, which acts as a gatekeeper between the mother and the fetus, promotes fetal growth and development and regulates the in utero environment through its waste exchange function [34]. In such a framework, air pollutants pose one of the most relevant threats to fetal development, as they may cause oxidative stress, systemic inflammation, and alterations in DNA methylation patterns in the placenta, ultimately affecting placental function and fetal reprogramming and thus potentially increasing the risk of reduced fetal growth, LBW, and prematurity [33,35,36]. Additionally, maternal air pollution exposure has been associated with hypertensive complications, i.e., preeclampsia and gestational hypertension, because of trophoblast invasion and increased vascular resistance, which may lead to the impairment of uteroplacental perfusion and eventually to maternal and fetal adverse outcomes [36]. Indeed, the fetus is particularly sensitive to exogenous harmful factors due to the high rate of cell proliferation characterizing fetal growth and rapid organ development [37]. Growing evidence supports the association between exposure to ambient air pollution, particularly PM, during pregnancy and the occurrence of multiple adverse outcomes [37,38,39,40]. The literature also suggests that ambient air pollution can be related to increased risk of some groups of congenital malformations, mainly CHD [21,41]. Some early studies have assessed the potential role of gestational exposure to outdoor air pollution in the first 3–8 weeks of pregnancy in increasing the risk of CHD [42,43,44]. However, although heart development begins between weeks 3 and 4 and the formation of the four chambers occurs by the end of week 7 of gestation, the window of susceptibility to environmental factors may not completely overlap with the time of heart development, and may even extend into the second and third trimesters of pregnancy [22,45,46].

In the following subsections, we will describe the main properties of the major air pollutants, the updated data regarding their possible involvement in the etiopathogenesis of CHD, and the biological processes underlying this association.

### 2.1. Particulate Matter

**Figure 1:**
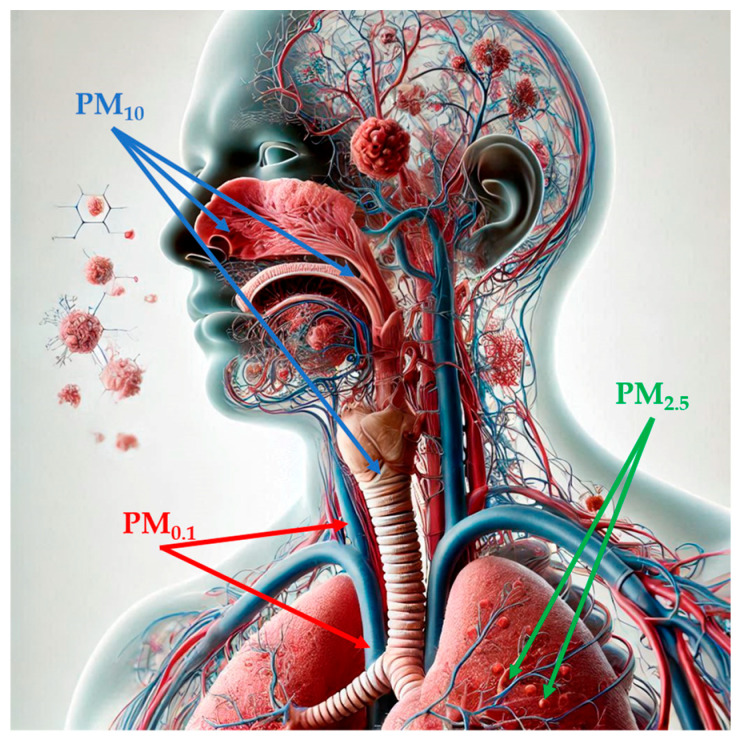
Penetration of particulate matter into the human body, depending on its size (image partially generated using the Artificial Intelligence tool Microsoft Bing). Abbreviations: PM1: particulate matter with aerodynamic diameter of less than 1 µM; PM2.5: par-ticulate matter with aerodynamic diameter of less than 2.5 µM; PM10: particulate matter with aer-odynamic diameter of less than 10 µM.

PM, a mixture of solid particles and liquid droplets, which presents high variability in size and may consist of hundreds of different chemicals, is considered the most reliable indicator of air pollution [17,47]. Notably, PM is classified according to its aerodynamic diameter: particles sized smaller than 10 µM in diameter (PM_10_), also called inhalable particles; PM_2.5_, known as fine particles; and PM_0.1_, or ultrafine particles, with a diameter, which is smaller than 0.1 µM [48]. Particle size is strongly correlated with health-related effects of PM since, with the decrease in size, particles may progressively deposit in the nasal, pharyngeal and laryngeal passages, trachea, and bronchi (PM_10_) in the respiratory bronchioles and alveoli (PM_2.5_) and, through the respiratory surfaces, in the systemic circulation (PM_0.1_) (Figure 1) [48,49,50,51]. PM_2.5_ can also originate directly from both natural sources, such as dust storms, volcanic eruptions, forest fires, and anthropogenic sources like inefficient fuel combustion and agricultural waste burning (primary PM_2.5_) or produced through physical (coagulation, condensation) and chemical processes involving pollutants such as NO_2_ and SO_2_ [47,48,52,53]. Increasing evidence has shown a risk association between short- and long-term exposure to ambient PM_2.5_ and PTB, mortality or incidence for cardiovascular and respiratory diseases, lung cancer, and type 2 diabetes [54]. In 2019, PM_2.5_-related deaths totaled nearly 4.2 million, an increase of 102.3% from 1990 to 2019, and with ischemic heart disease, stroke, and chronic obstructive pulmonary disease as the three leading causes of death [54]. According to the 2021 WHO Air Quality Guidelines, the recommended annual and daily average levels for PM_2.5_ should not exceed 5 and 15 µg/m^3^, respectively [55]. As for PM_10_, the WHO recommendation corresponds to an annual level of 15 µg/m^3^, based on the assessment of the long-term effects of PM_10_ (i.e., mortality) and not considering that a large proportion of PM_10_ consists of PM_2.5_ [56]. Importantly, in 2022, most of the European urban population was exposed to concentrations of PM_2.5_ (96%) and PM_10_ (83%) above WHO guidelines, despite ongoing emission reduction policies [57].

#### 2.1.1. The Association Between Maternal Exposure to Particulate Matter and Congenital Heart Disease

So far, numerous case–control and cohort studies have explored the association between maternal exposure to PM and the risk of CHD, providing conflicting results. A Chinese case–control study involving a total of 5213 infants (1039 with birth defects and 4174 without congenital anomalies, including live births and stillbirths) showed that, for every 10 µg/m^3^ increase in concentration, maternal exposure to PM_2.5_ (daily average concentration of ambient air pollutants obtained from 22 air quality monitoring stations in the period 2015–2019, which included the period of maternal pregnancy) during the second and third trimesters of gestation was associated with a higher risk of CHD (Odds Ratio—OR = 1.228, 95% Confidence Interval—95%CI: 1.141–1.322, *p* < 0.001 and OR = 1.236, 95%CI: 1.154–1.324, *p* < 0.001, respectively), after adjusting for confounders [46]. No significant associations were instead observed between PM_10_ and CHD at any time during pregnancy [46]. However, this study could not evaluate the mobility during the gestational period, resulting in potential exposure misclassification. Furthermore, it was not possible to estimate maternal exposure at the address of residence and/or work, but the survey was only based on the average value measured in the whole city [46]. Finally, the exclusion of pregnancy termination before week 28 of pregnancy could have led to an underestimation of CHD prevalence [46]. A nationwide surveillance-based case–control study performed in China including 1,434,998 births and a total of 7335 CHD (live births or stillbirths born at ≥28 weeks of gestation) from January 2014 to September 2017 explored the effect of maternal exposure to PM_2.5_ (calculated by assigning the satellite monthly mean concentration on the geocoded residential address of each participant) on CHD onset before and after conception [7]. The authors reported a small increase in CHD risk (OR = 1.02, 95%CI: 1.00–1.05) per each 10 µg/m^3^ increase in maternal exposure to PM_2.5_ during the periconceptional period, with a slightly more pronounced effect three months before conception (OR = 1.03, 95%CI: 1.01–1.05) compared with the first trimester of pregnancy (OR = 1.02, 95%CI: 1.00–1.04) [7]. This indicates that, in addition to the window of susceptibility for cardiac development, the accumulation of a high concentration of pollutants three months before conception, where the preantral follicle gradually mature in the ovary until ovulation, is equally critical for potential teratogenic effects to the embryo [7]. Considering CHD types, the only significantly positive association was observed for septal defects (OR = 1.04, 95%CI: 1.01–1.08), with an attributable risk proportion of 8.44% during periconception [7]. In addition, for every 10 µg/m^3^ increase in PM_2.5_, the risk of CHD increased by 3% for mothers younger than 35 years (*p* = 0.039) and by 9% for those living in areas with low disposable income per capita, the latter being likely to have less access to medical services and less ability to reduce their exposure to air pollution [7]. Another Chinese retrospective case–control study collecting air pollution data from 39 monitoring stations in 2019 included a total of 1208 children with birth defects, 607 of which were CHD, taken from 69,995 newborns [58]. The study reported a slight increase in CHD risk in relation to every 10 µg/m^3^ increase in PM_2.5_ in the first (OR = 1.05, 95%CI: 1.02–1.09) and third month (OR = 1.04, 95%CI: 1.01–1.08) of gestation after adjusting for confounders, consistent with the findings of Yuan et al. [7,58].

Based on a recent systematic review and meta-analysis including 32 studies (21 case–control and 11 cohort studies, for a total of more than 7 million births in the years 2002–2022) evaluating 6 pollutants and 11 subtypes of CHD, no significant associations were found with an overall or any subtype of CHD for continuous exposure to PM_2.5_, except for a borderline association with the transposition of great arteries (TGA) (OR = 1.14, 95%CI: 1.00–1.31 per each 10 µg/m^3^) [15]. Similar results were also obtained in the categorical analysis, where high versus low levels were compared [15]. Conversely, maternal exposure to PM_10_ was significantly associated with a higher risk of overall CHD in both continuous (OR = 1.03, 95%CI: 1.01–1.05) and categorical analyses (OR = 1.04, 95%CI: 1.00–1.09), but not with any CHD subtypes [15]. Thus, the data provided only suggest a significant association of CHD risk exclusively with exposure to PM_10_ [15]. However, it is important to note that the same authors, in a retrospective review of time-series studies aimed at assessing the short-term effects of ambient air pollution of CHD occurrence, showed that the most susceptible windows to PM were mainly located within the second and the third trimesters of pregnancy [15]. Heterogeneity in the period of exposure considered, along with differences in the methods of pollutant measurement, the concentration and chemical compositions of pollutants, study designs, and demographic features of populations, may have produced inaccuracies in exposure estimates [15]. Finally, an umbrella review embracing eleven systematic reviews (eight with meta-analyses) published between 2011 and 2021, three of which exclusively evaluated the exposure to PM while four systematic reviews reported data on all measured pollutants, provided mixed results on the relationship between prenatal exposure to PM and CHD [59]. In particular, results from three meta-analyses were suggestive of a significantly positive association between prenatal exposure to PM_10_ with ASD and of increased risk of tetralogy of Fallot (ToF) in relation to prenatal exposure to PM_2.5_ [59]. Higher statistical heterogeneity of effect estimates were reported for associations between PM_10_, PM_2.5_, and ASD, VSD, and conotruncal defects, PM_10_ and coarctation of aorta (CoA), and PM_10_ and PDA [58]. Additionally, the paucity of data can be explained by imprecision in the assessment of associations between PM_2.5_, ASD, VSD, and ToF [59].

Overall, there is moderate evidence for the association of maternal exposure to PM, particularly PM_2.5_, with CHD risk, while associations of PM with certain CHD subtypes are rated as having low or very low certainty. The substantial differences between systematic reviews not only reflect the heterogeneity of studies in terms of populations, study design, exposure assessment methods, periods of exposure, and confounding factors, but also probably depend on the inclusion criteria and the number of studies included, which in turn influence the reliability of meta-analytical results (Table 1). Therefore, future multicenter cohort studies should be based on a larger sample size and standardized methods of exposure measurements to ensure more robust and comparable results.

#### 2.1.2. The Relationship Between Particulate Matter and Congenital Heart Disease: Biological Mechanisms

A series of mechanisms have been postulated to support the potential association of prenatal exposure to PM with CHD. Experiments in cell cultures and rodent models exposed to PM, although having provided relevant information for the identification of cellular pathways implicated in PM toxicity, cannot be directly translated to humans due to differences in PM exposure levels (for cells) and in breathing and filtering patterns and nasal anatomy (for animals) [51]. On the other hand, controlled exposure studies conducted in humans may generally measure exposure over a few hours and are thus unable to evaluate the effects of chronic exposure [51]. A limited number of studies have explored the consequences of gestational exposure to PM on the cardiovascular system, although, as described in Section 2, the altered functioning of the placenta, induced by air pollution, has been recognized to contribute to adverse health outcomes in early and late life [60,61]. Therefore, any environmental event occurring during gestational age can potentially be responsible for alterations of fetus development.

Listed below are the main proposed processes that could explain the link between PM exposure and the onset of CHD:

1. Oxidative stress plays a primary role in mediating systemic responses triggered by PM from the initial locus of the airways and lungs, including cardiovascular outcomes [51,62]. In addition to containing reactive oxygen species (ROS, including superoxide radicals, hydrogen peroxide, and hydroxyl radicals), PM may lead to the overproduction of endogenous ROS through redox reactions of the particles with sensory receptors on alveoli surfaces [62,63,64]. Alternatively, upon achieving access to the bloodstream, PM may interact with vascular cells in the endothelium, resulting in the disruption of cellular antioxidant signaling [65]. Exposure to PM_2.5_ can promote the increased expression of NADPH oxidase 4 (Nox4), the most important Nox isoform in the heart, which, by transferring an electron to molecular oxygen, is considered to be among the main sources of oxidative stress [60,66]. Oxidative stress, promoting lipid peroxidation and protein oxidation, causes systemic inflammation, which in turn can be responsible for the development of endothelial dysfunction, atherosclerosis, up to the onset of cardiac dysfunction with arrhythmia and heart rate variability, cardiac remodeling and heart failure, and thrombosis [63,64]. In particular, an excess of superoxide radicals may interact with nitric oxide, generating peroxynitrite, a reactive intermediate that, in addition to producing DNA strand breaks and lipid membrane damage, can bind to tyrosine residues of proteins, giving rise to 3-nitrotyrosine (3-NTp), considered a stable product of protein nitration and a reliable biomarker of nitrosative stress, which is in turn associated with pathological pregnancies [64]. Interestingly, placental levels of 3-NTp have been shown to increase by 35% for each interquartile range (IQR) increment in PM_2.5_ over the entire pregnancy, therefore posing a risk for the developing fetus [65]. In addition, within the same birth cohort study, each IQR increment in PM_10_ exposure during the first and second trimesters of pregnancy was associated with increased levels of mitochondrial 8-hydroxy-2′-deoxyguanosine (8-OHdG, a marker of DNA oxidative damage) in umbilical cord blood of 23% and 16.6%, respectively, while no significant associations were found with exposure to PM_2.5_, suggesting that the early- and mid-pregnancy are potentially harmful for adverse birth outcomes [67,68]. Recently, research conducted on a cohort of 305 American pregnant women documented increased urinary 8-OHdG levels during the second trimester in relation to one IQR increase in the average cumulative PM_2.5_ concentration in the 3–7 days before urine collection, indicating that the rapid growth of the placenta and fetus can contribute to the marked increase in oxidative stress levels [68]. In the same cohort, each IQR increase in the PM_2.5_ concentration in the first trimester was also associated with an 8.2% increment in malondialdehyde, a marker of lipid peroxidation, in the first trimester, whereas no associations were found in the other two trimesters [68].
2. A number of studies have explored the association between gestational exposure to PM_2.5_ and placental DNA methylation, which, by controlling key genes involved in the regulation of cellular placental processes, is essential for the physiological fetal growth [69]. In utero exposure to PM_2.5_ in rodent models triggers cardiac dysfunction by inducing a decrease in the expression of sirtuin (Sirt) 1 and Sirt2, NAD^+^-dependent histone deacetylases, and an increase in DNA methyltransferase (Dnmt) 1, Dnmt3a, and Dnmt3b [70]. Conversely, Dnmt1 downregulation, no changes in Dnmt3a protein expression, and increased expression of Sirt1 and Sirt2 were observed in myocardial tissues of offspring following preconceptional exposure to PM_2.5_ [71]. The methylation status of leptin, a hormone that not only regulates energy homeostasis but that is also produced by placental trophoblasts, and which has a functional role in embryo implantation, intrauterine development, and fetal growth, is inversely related to an IQR increase in PM_2.5_ exposure during the second trimester of gestation; 3-NTp acts as a mediator of this association, doubling its placental concentration [72,73]. Furthermore, prenatal exposure to PM_2.5_ was significantly associated with epigenetic modifications in selected placental DNA repair genes (i.e., *APEX1*, *OGG*, and *ERCC4*) and tumor suppressor genes (i.e., p53, *DAPK1*), suggesting the potential of these pollutants to interfere with the fetal and neonate repair ability [74]. *APEX1* and *DAPK1* appear to exert protective effects in cardiac ischemia–reperfusion injury by counteracting oxidative damage [75,76]. On the other hand, *OGG1* has been shown to play a crucial role in cardiac development in zebrafish, while *OGG1* loss has been associated with severe cardiac morphogenesis and functional abnormalities [77]. Zhao et al. [69] recently reported significant association of PM_2.5_ exposure with changes in DNA methylation of candidate genes implicated in the regulation of the cell cycle and energy metabolism, including *BID* and *IGF-2* (a gene encoding the most important mitogen for cardiomyocytes during fetal growth, [78]) during the entire pregnancy and *FOXN3* (a gene implicated in cell cycle and transcription regulation at the cellular level and also in the development of the interatrial septum and trabeculae in frog hearts [79,80]) during the second trimester. Of note, methylation of *BID*, a gene critical for developmental apoptosis and playing a pivotal role in both myocardial infarction with reperfusion and heart failure [81], might explain approximately 30% of PM_2.5_ effect on the fetal head growth characteristics occurring during the second trimester [69]. As for PM_10_, higher levels of exposure during the first trimester were associated with a 1.8% decrease in DNA methylation of a placental long interspersed nucleotide element (*LINE*)-1, which is frequently used as a proxy of global methylation in newborns with fetal growth restrictions [82]. On the other hand, placental DNA methylation in *HSD11B2*, a gene involved in glucocorticoid metabolism and having a central role in fetal growth, increased by 1% and 2.3% for each 10 μg/m^3^ increment of exposure to PM_10_ in the first and second trimester, respectively [82]. Maternal PM_10_ exposure in the third trimester and throughout pregnancy was also associated with the methylation levels of cord and maternal blood *H19* differential methylation region, known to be related to health outcomes [83,84]. Finally, significant negative associations were found between every 10 µg/m^3^ increment of maternal PM_10_, PM_2.5_, and PM_1_ exposure with cord blood *LINE-1* methylation and a significantly inverse association was found between PM_1_ concentration and maternal *LINE-1* methylation level [85]. Importantly, a decrease in the methylation level of *LINE-1*, resulting in increased expression or decreased degradation of *LINE-1* DNA or transcripts, may promote immune system response and, consequently, inflammation [86].
3. As mentioned in item 1, inhalation exposure to PM_2.5_ can lead to cardiac remodeling, which consists of structural changes such as fibrosis and hypertrophy both in cardiomyocytes and the connective tissue surrounding the heart and is characterized by an imbalance of extracellular matrix production and degradation, ventricular morphologic alterations, and decreased contractility [60,70,71]. PM_2.5_-induced oxidative stress during parental preconception and gestation triggers the activation of myocardial inflammatory response, with increased release of pro-inflammatory cytokines, such as interleukin (IL)-1β, IL-6, IL-8, IL-15, tumor necrosis factor alpha (TNF-α), E-selectin, P-selectin, and C-reactive proteins (CRPs) [66,72,87]. IL-6, a crucial cytokine in cardiac pathogenetic processes, in addition to being regulated at transcriptional and posttranscriptional levels, is also secreted by nuclear factor kappa-light-chain-enhancer of activated B cells (NF-κB), which in turn is activated by IL-6 and TNF-α [87,88]. The persistent dysregulation of IL-6 synthesis may cause chronic inflammation and autoimmunity and, by inducing the upregulation of the vascular endothelial growth factor, also promotes angiogenesis and vascular permeability [87]. CRP levels, which are suggestive of low-grade systemic inflammation, may increase during pregnancy in response to both infectious and non-infectious environmental determinants, including air pollutants [89]. Indeed, as revealed by a population-based cohort study in the Netherlands, exposure to the third and fourth PM_10_ quartiles in early pregnancy was associated with an increase in maternal blood CRP levels by 8 and 32%, respectively, while the exposure to the highest PM_10_ quartile during the entire pregnancy was related to increased fetal cord blood CRP levels at delivery by over 200% [88]. Elevated levels of inflammation are also associated with the upregulation of fibrogenic mediators such as collagen to maintain optimal cardiac functioning [70,71].

A summary of potential actions of PM leading to increased occurrence of CHD is reported in Table 2.

### 2.2. Nitrogen Dioxide and Sulfur Dioxide

NO_2_, a reddish-brown gas commonly generated from the high-temperature combustion of fuels in processes linked to transportation, industry, and heating, belongs to a group of highly reactive gasses known as nitrogen oxides, of which it is considered the main indicator [17,90]. Like other nitrogen oxides, NO_2_ can also react with other chemicals to give rise to PM and O_3_ [88]. When inhaled, high concentrations of NO_2_ may contribute to the development or worsening of existing respiratory diseases, such as asthma, particularly in children and the elderly, exacerbating respiratory symptoms and increasing the risk of hospitalization [90]. In 2020, vehicle traffic was the main contributor (37%) to NO_2_ emissions in Europe and, despite a drop in emissions by 48% over the years 2005–2020, chronic exposure to NO_2_ above WHO guideline levels of 10 µg/m^3^ per year was estimated to have caused 52,000 premature deaths in 2021 [25,55,91]. Furthermore, it has been estimated that, in 2022, 88% of the European population living in urban areas was exposed to NO_2_ levels exceeding WHO guidelines [57]. A number of studies also indicated a relationship between long-term exposure to NO_2_ and increased incidence of cardiovascular disease [92,93], while exposure to NO_2_ during pregnancy has been reported to be significantly associated with LBW and PTB [94,95].

SO_2_, a colorless gas belonging to the broader class of sulfur oxides which are detectable in atmosphere at concentrations lower than those of SO_2_, is mainly generated by the combustion of fossil fuels by power plants (responsible for around 40% of SO_2_ emissions in Europe in 2020) and other industrial facilities and, to a lesser extent, by the smelting of mineral ores and volcanic emissions [17,25,96]. Short-term exposure to SO_2_ is positively associated with all-cause and respiratory mortality; however, the adverse effects of SO_2_ inhalation in humans also include cardiovascular disease, disorders of the nervous system, and type 2 diabetes [97,98]. Regardless of the exposure window, long-term exposure to ambient SO_2_ during gestation also appears to increase the risk of PTB and SGA [20,99]. Of note, maternal exposure to SO_2_ in early pregnancy has been recently positively associated with the risk of omphalocele, one of the most frequent congenital defects of the abdominal wall [100]. The latest WHO air quality guidelines recommend SO_2_ concentrations of 40 µg/m^3^ on a 24 h average and 500 µg/m^3^ on a 10 min average, while a 2008 European Directive set two limit values to protect human health from the effects of SO_2_, i.e., that the hourly mean value that cannot exceed 350 µg/m^3^ more than 24 times in a year and that the daily mean value that cannot exceed 125 µg/m^3^ more than 3 times in a year [55,101]. Notably, the current SO_2_ levels in Europe do not exceed 4 ppb, corresponding to levels 4 times lower than those established by the WHO guidelines [102].

#### 2.2.1. The Association Between Maternal Exposure to Nitrogen Dioxide and Sulfur Dioxide and Congenital Heart Disease

Currently, there is no consistent evidence on the actual role of NO_2_ and SO_2_ in influencing the likelihood of developing CHD in the offspring. The above-mentioned study by Sun Et Al. [46], including data on babies born between 28 weeks of gestation and 7 days after birth (N = 5213), revealed that the risk of CHD significantly increased for every 1 µg/m^3^ increment of NO_2_ exposure during the first trimester (OR = 1.318, 95%CI: 1.210–1.435, *p* < 0.001), whereas it was inversely correlated with SO_2_ over the entire period of pregnancy (*p* < 0.001). The study by Huang Et Al. [58], which analyzed the relationship between ambient air pollution and the occurrence of birth defects including CHD between 2019 and 2020, documented a small effect of NO_2_ exposure throughout pregnancy on the increased risk of CHD [58]. On the other hand, each 10 µg/m^3^ increase in SO_2_ during the first and second months of pregnancy was associated with a 34% and 46% increased risk of CHD, respectively, in the adjusted single-pollutant model, although these associations become non-significant in the multi-pollutant model [58]. A subsequent meta-analysis (24 and 19 studies published from 2002 onwards investigating the relationship between NO_2_ and SO_2_ exposure, respectively) showed no significant association of gestational NO_2_ exposure with either overall CHD or CHD subtypes [15]. At the same time, non-significant negative associations with maternal NO_2_ exposure were also observed in both continuous and categorical analyses in some of the included studies [15]. Conversely, significantly inverse associations were found between SO_2_ and risk of VSD in the continuous exposure analysis (OR = 0.95, 95%CI: 0.91–0.99) and for ToF in the categorical exposure analysis (OR = 0.93, 95%CI: 0.63–0.99), while no significant association was detected with the overall CHD group [15]. According to the time-series analysis conducted by the same authors that aimed to explore the most susceptible windows for pollutants, the maximum effect for both NO_2_ and SO_2_ appears to be concentrated mainly in the mid- and late-pregnancy [15]. However, as previously reported, exposure estimates were subject to imprecision, as they were not based on the geocoded residential address, but on local monitoring stations, while, due to insufficient information, it was not possible to perform CHD subtype analyses that adjusted for risk factors [15]. Within an umbrella review, Michel and co-authors [59] reported a positive association of prenatal NO_2_ exposure with CoA (found in four meta-analyses) and with ToF (observed in two meta-analyses), as well as null associations between NO_2_ and overall CHD, ASD, VSD, and ToF (the latter association was shown in two meta-analyses). As for SO_2_, a significantly positive association was found between CoA and ToF (in one meta-analysis) and with CoA in relation to categorical exposure (in one meta-analysis) [59]. It is important to note that the risk of bias (including data sources, systematic search strategies, and lack of risk of bias assessments) may have influenced risk estimates of SO_2_ exposure with the occurrence of ASD, VSD, and CoA [59]. Furthermore, high statistical heterogeneity was reported for associations between SO_2_ and CoA and ToF and between NO_2_ and ASD, VSD, and ToF [59].

In summary, except for moderate evidence for a greater risk of CoA related to prenatal exposure to NO_2,_ the relationship of NO_2_ and SO_2_ exposure with overall CHD and certain CHD subtypes remains inconclusive due to low precision and high statistical heterogeneity between systematic reviews. In particular, the results on the association between SO_2_ and the onset of CHD are extremely conflicting, and no conclusion on the real effects of SO_2_ exposure during pregnancy can be drawn. There might be signals of association between maternal exposure to NO_2_ with overall CHD and ToF, but these findings need to be confirmed in future studies, possibly evaluating multi-pollutant exposures and applying standardized methods in exposure assessment and case definition (Table 3).

#### 2.2.2. The Relationship Between Nitrogen Dioxide, Sulfur Dioxide, and Congenital Heart Disease: Biological Mechanisms

As reported in the previous sections, the placental tissue represents an ideal target for ambient air pollutants, as it is subject to various biological mechanisms such as oxidative/nitrosative stress, inflammation, and epigenetic changes, which overall contribute to disrupt the mother–fetus axis and compromise fetal growth [103]. Therefore, we report below the possible processes through which NO_2_ and SO_2_ can be involved in the development of CHD (see also Table 4):

1. Within the ENVIRONAGE study, a Belgian birth cohort study enrolling 330 mother–newborn pairs, maternal NO_2_ exposure was positively and significantly correlated with placental 3-NTp over the entire pregnancy (see Section 2.1.1), although the association became non-significant after adjustments for covariates. Notably, although nitration of placental proteins is also detected in normal pregnancies and attacks only a limited number of proteins, including those involved in trophoblast invasion and the regulation of placental vascular reactivity, at higher levels it may cause dyshomeostasis of the placental function [65].
2. Changes in mitochondrial DNA (mtDNA) content can also be considered to be a mediator linking air pollution to fetal growth restriction [104]. Indeed, data from two European cohorts in Belgium and Spain showed that an increase of 10 µg/m^3^ in NO_2_ exposure during pregnancy was associated with both a significant decrease in placental mtDNA content and birth weight, while, on the other hand, the amount of mtDNA in the placenta was positively correlated with weight at birth [104]. mtDNA is multi-copy, and, while its abundance is relatively stable in physiological conditions with only small fluctuations, it may greatly vary depending on certain pathogenic factors, such as cellular redox imbalance [105]. Therefore, damage to mitochondria, which are particularly susceptible to the effects of environmental chemicals due to their lack of repair ability, and the subsequent oxidative stress may cause a nutrient deficit and mediate the biological effects of prenatal exposure to NO_2_ on LBW [105].
3. As reported for PM, NO_2_ exposure may promote increased inflammation. A population-based cohort study performed in the Netherlands on 6508 mother–infant pairs reported a positive association between increased NO_2_ exposure levels (third and fourth quartiles) during total pregnancy and levels of fetal cord blood CRP at delivery (>1 mg/L) with a monotonic increase (2.85 times and 3.42 times, respectively, compared to the first NO_2_ quartile) [89]. However, unlike PM_10_, the same study did not observe any significant association between NO_2_ exposure in the first trimester of pregnancy and maternal blood CRP levels [89].
4. In the previous sections, we have shown that air pollution can be associated with adverse birth outcomes by inducing epigenetic modifications that can have a substantial impact during embryogenesis, being linked to differential protein expression, including proteins involved in antioxidant defense [106]. During embryogenesis, DNA methylation is a dynamic process in which DNA can undergo a series of methylation and demethylation steps, and is, therefore, particularly vulnerable to environmental stimuli [83]. A large-scale meta-analysis aimed at investigating the association between NO_2_ exposure during gestation and epigenome-wide DNA methylation in newborns found different methylation patterns in cord blood genes implicated in mitochondrial function and differential methylation and expression in genes playing key antioxidant roles, i.e., catalase and thyroid peroxidase, in relation to maternal NO_2_ exposure [106]. Furthermore, a study on 527 mother–infant pairs exploring the association between prenatal exposure to air pollution, *H19* methylation patterns (see Section 2.1.1), and birth weight and length found that NO_2_ exposure during the entire pregnancy and first and third trimesters was positively correlated with the methylation level in the *H19* promoter region in cord blood [83]. Conversely, a negative association was detected between SO_2_ exposure during the entire pregnancy and the first trimester and *H19* promoter region methylation level [83]. Although birth sizes were both correlated with maternal SO_2_ (significant decrease) and NO_2_ exposure (significant increase), the mediating effect of *H19* methylation status in this relationship was not observed [83].

**Table 4:** Possible biological mechanisms underlying the relationship between exposure to nitrogen and sulfur dioxide and the risk of congenital heart disease.

NO_2_ Effects	Reference	SO_2_ Effects	Reference
Significantly positive association between NO_2_ exposure during the entire gestation and placental 3-NTp	[65]	Negative association of SO_2_ exposure in the first trimester and throughout pregnancy with the methylation level of the H19 promoter region	[83]
Gestational NO_2_ exposure significantly and negatively association with placental mtDNA content during the entire pregnancy	[104]		
High levels of NO_2_ during the entire pregnancy positively associated with fetal cord blood CRP levels at delivery	[89]		
Maternal NO_2_ exposure correlated with differential methylation and expression of CAT and TPO in cord blood during gestation	[106]		
Positive association of NO2 exposure in the first and third trimesters and the entire pregnancy with *H19* methylation pattern in cord blood	[83]		

Abbreviations: 3-NTp: 3-nitrotyrosine; CAT: catalase; CRP: C reactive protein; mtDNA: mitochondrial DNA; NO_2_: nitrogen dioxide; SO_2_: sulfur dioxide; TPO: thyroid peroxidase.

### 2.3. Ozone and Carbon Monoxide

In contrast to stratospheric O_3_, occurring in the Earth’s upper atmosphere to form a protective layer against ultraviolet radiation, tropospheric O_3_ is a harmful air pollutant, not directly emitted into the atmosphere, but generated by interactions between nitrogen oxides and volatile organic compounds (VOC) in the presence of sunlight [17,107]. O_3_ is one of the major constituents of photochemical smog, reaching the highest levels in colder months and in urban areas, although, thanks to the possibility of being transported over long distances, it can also be detected at high concentrations, even in rural areas [17,107]. In 2021, 22,000 premature deaths were attributed to acute exposure to O_3_, which, according to the global update to WHO recommendations, should not exceed the average daily maximum 8 h mean concentrations of 100 μg/m^3^ during short-term exposure (defined as the 99th percentile of the distribution of daily values and equivalent to 3–4 exceedance days per year) [55,108,109]. Conversely, the long-term guideline sets a maximum value of 60 μg/m^3^ as the average of daily maximum 8 h mean O_3_ concentrations during the peak season (defined as the six consecutive months of the year with the highest levels of O_3_) [109]. Anyway, in 2022, almost all of the urban population (94%) in Europe was exposed to O_3_ concentrations above the short-term guideline level [57]. A large body of evidence supports the increased risk of respiratory health outcomes in terms of mortality and exacerbations of symptoms in relation to short-term O_3_ exposure [110]. In addition, emerging data indicate a possible role of long-term exposure to O_3_ in increasing the risk of respiratory and cardiovascular mortality [109]. Notably, in addition to a higher risk of PTB-linked increased O_3_ exposure during pregnancy, especially during the first and second trimester [111,112], O_3_ exposure before and during pregnancy has been associated with congenital malformations, including defects of the circulatory system [113].

CO, a colorless, odorless, and tasteless gas resulting from the incomplete combustion of carbonaceous fuels (e.g., petrol, charcoal, and natural gas) has its main sources in outdoor environments in vehicles [17,114]. Compared to indoor settings, where CO may cause dizziness and unconsciousness up to death due to its binding to hemoglobin with an affinity 200 to 240 times that of oxygen, thus reducing blood oxygenation, elevated levels of CO rarely occur outdoors [114,115]. The latest WHO guidelines on ambient air quality established a maximum CO value of 30 mg/m^3^ for 1 h exposure, decreasing to a maximum of 10 mg/m^3^ over the 8 h daily average [55]. It should be noted, however, that following improvements in transportation efficiency, such as the installation of catalytic converters on vehicles, CO emissions in the EU decreased 7.2 percent in 2022 and by approximately 70% since 1990 [116]. Acute CO exposure can impact the cardiovascular system with a higher risk of developing arrhythmia [114], hospital admissions [117], and mortality for cardiovascular disease, especially coronary heart disease [118,119]. In addition, short-term exposure to ambient CO during pregnancy augmented the occurrence of PTB in newborns, while short- and long-term exposure to low CO concentrations seem to give a protective effect against preterm and very preterm births [120].

#### 2.3.1. The Association Between Maternal Exposure to Ozone and Carbon Monoxide and Congenital Heart Disease

As already observed for other pollutants, despite the large overall body of research, there is currently no complete evidence on the role of O_3_ and CO in the development of CHD. The above-mentioned study by Sun Et Al. [46], investigating the association between maternal exposure to ambient air pollutants per trimester of pregnancy and the risk of CHD, surprisingly revealed a lower occurrence of CHD related to each 10 µg/m^3^ increase in CO exposure during the whole pregnancy, particularly in the late phase (OR = 0.983, 95%CI: 0.977–0.990, *p* < 0.001), similarly to SO_2_, although with a smaller effect [46]. Within another case–control study, Huang Et Al. [58] found a borderline excess prevalence of CHD for every 10 µg/m^3^ of exposure to CO and O_3_ during the second month of gestation (OR = 1.004, 95%CI: 1.001–1.007 and OR = 1.024, 95%CI: 1.005–1.043, respectively, in the adjusted single-pollutant model). The two associations maintained statistical significance also in the multi-pollutant model, with an increase in CHD risk of 0.8% and 2.8% related to CO and O_3_ exposure during the second and third months of pregnancy, respectively [58]. A recent meta-analysis of 21 and 14 studies evaluating O_3_ and CO exposure, respectively, and not including [46,58], reported only a marked increase in ToF risk in relation to both continuous (OR = 2.25, 95%CI: 1.42–3.56) and categorical CO exposure (OR = 1.24, 95%CI: 1.01–1.54), while no significant associations were observed with gestational O_3_ exposure [15]. Furthermore, based on a time-series analysis, the first weeks of pregnancy appear to be the most sensitive to the effects of CO and O_3_ [15]. According to the umbrella review by Michel et al. [59], an inverse association between prenatal exposure to CO and ASD risk was reported in two meta-analyses, while one meta-analysis showed a positive association between CO exposure and ToF and O_3_ exposure and ASD occurrence in offspring. The authors also highlighted imprecise estimates and publication biases in the assessments of correlations between CO exposure, conotruncal defects, and ASD [59]. Two recent studies, both conducted in China, investigated the association between maternal exposure to O_3_ and the risk of CHD. The first of them was a case-crossover study including 6661 children with diagnoses of birth defects (pregnancies occurring between March 2017 and September 2020), assessing O_3_ exposure at the individual level by cross-referencing the latitude and longitude of maternal residence with daily measurements of near-surface O_3_ [113]. O_3_ exposure in the periconceptional period (the first trimester before and after conception) was associated with a higher risk of birth defects, which reached an increase of 4.2% for each 10 µg/m^3^ increment of O_3_ during the first month before pregnancy [113]. Except for the second and third month of gestation, the authors also observed an elevation in CHD occurrence, with the maximum achieved during the second month before pregnancy (OR = 1.037, 95%CI: 1.021–1.053, *p* < 0.001) [113]. This finding indicates that the three months before pregnancy represent a critical period for CHD development, since oocyte quality can be affected, although heart and cardiac vessel formation occurs at the beginning of pregnancy [113]. Within a cross-sectional study that enrolled a total of 27,817 pregnant women from 2013 to 2021 (N = 4842 with children with CHD, N = 22,975 control mothers) and that assessed O_3_ exposure at the mothers’ residential address at different stages of pregnancy, Wang et al. [121] showed a significant increase in risk of pooled CHD (OR = 1.127, 95%CI: 1.098–1.155) associated with an increment of 10 µg/m^3^ in O_3_ exposure during the embryonic period (weeks 3–8 of gestation). O_3_ exposure in the same period of gestation also caused an increased risk for VSD (17%), ToF (19%), TGA (17%), pulmonary stenosis (17%), pulmonary atresia (12%), and persistent left superior vena cava (11%) [121]. Of note, the estimates monotonically increased with increasing exposure, except for TGA, and the effect augmented in the warm season compared to the cool season, since, in the warm season, O_3_ concentration generally increases, while the high temperatures and humidity make the respiratory pathway more vulnerable to the effects of air pollutants [121]. Additionally, considering different windows of exposure, a stronger association was found during periconception (three months before the last menstrual period until 13 weeks of pregnancy) compared with during the first trimester (1–13 weeks of gestation) and preconception (3 months before the last menstrual period), and the estimates remained unchanged even after adjustment for other pollutants (PM_2.5_, NO_2_), maternal smoking, and alcohol consumption [121]. The authors also underscored that mothers in the CHD group had a lower socioeconomic status compared to those in the control group, which is linked to limited access to healthcare, lower health knowledge, and greater psychosocial stress, all factors contributing to an increased risk of CHD [121].

Therefore, these results collectively support a potential effect of O_3_ in increasing the risk of CHD occurrence in offspring. In particular, two recent studies, which were based on a large sample size, have shown a robust association between O_3_ exposure, even during preconception time, and higher risk of pooled CHD and some CHD subtypes. In contrast, the relationship between maternal exposure to CO and CHD risk remains currently uncertain due to imprecise estimates (Table 5). Further cohort studies, based on accurate exposure measurements performed at different pregnancy times, could finally establish temporal relationships and make causal inferences in the relationship between O_3_ and CO and the onset of CHD.

#### 2.3.2. The Relationship Between Carbon Monoxide, Ozone, and Congenital Heart Disease: Biological Mechanisms

A series of mechanisms have been proposed to attempt to explain the possible association between exposure to ambient O_3_ and the onset of CHD, and, despite the current lack of evidence on the actual role of CO in increasing CHD risk, CO also displays toxic cellular effects potentially related to adverse outcomes in pregnancy (see the summary of effects in Table 6).

1. Once it has entered the lungs, O_3_ stimulates the intracellular and extracellular accumulation of ROS [122]. Mitochondria represent the most relevant site for cellular ROS production; thus, any process affecting their integrity can promote further generation of ROS [122]. Mice chronically exposed to O_3_ exhibited mitochondrial dysfunction reflected by decreased mitochondrial membrane potential, increased mitochondrial oxidative stress, and reduced expression of mitochondrial complex I, III, and V of lung bronchioles [123]. Conversely, acute O_3_ exposure resulted in reduced mitochondrial complex I, III, and V expression in mice lungs [124]. Therefore, O_3_ appears to modify the expression of mitochondrial complexes, potential sources of ROS, in addition to acting directly on macromolecules, producing the oxidation of lipids, proteins, and DNA, and ultimately leading to cell and tissue damage [122,125,126]. Mitochondria, especially the electron transport chain, also represent a major target for CO, which may directly and significantly inhibit cytochrome C oxidase (belonging to the complex IV), as documented in human muscle tissues [127]. Alternatively, in rat brains, the hypoxia resulting from increased blood CO concentration leads to reduced activity of the cytochrome chain, blocking energy production in oxidative phosphorylation and thus resulting in increased ROS due to electron loss in the electron transport chain [128,129]. An experimental study on neuronal brain cultures [130] also showed that CO is able to induce ROS production firstly from mitochondria (in the initial minutes of exposure), then through the activation of xanthine oxidoreductase, which produces ROS as a byproduct of conversion oxidation of xanthine to uric acid [131], and, with the most marked effect, in the post-CO exposure, due to the activation of NOX (primarily located in endosomes and representing a major source of ROS; see Section 2.1.1) [132]. In addition, CO exposure promotes lipid peroxidation and causes a significant decrease in glutathione levels in both neurons and astrocytes compared to untreated cells [129].
2. The loss of redox balance due to chronic exposure to O_3_ is, in turn, responsible for local inflammation [122,125]. Indeed, O_3_ may induce the secretion of cytokines and inflammatory factors including NF-κB, a transcription factor playing a central role in various cellular processes including pro-inflammatory responses, TNF-α, and IL-6 in both the lungs and cerebral cortex [122,133]. Notably, exposure to O_3_ in rats resulted in an increased macrophage infiltration into epicardial adipose tissue, a source of multipotent stem cell populations whose inflammation can be predictive of increased cardio-metabolic risk, and perirenal adipose tissue [134,135]. In addition, O_3_ exposure was associated with a significant upregulation of pro-inflammatory genes, i.e., *TNF-α* and genes encoding monocyte chemoattractant protein-1 (a chemokine regulating migration and infiltration of macrophages, [136]) and leptin (a hormone acting as a master regulator of energy balance and body adiposity [137]) and with increased expression of inducible nitric oxide synthase, which contributes to inflammation and oxidative stress [134]. At the same time, the authors observed the downregulation of genes encoding the anti-inflammatory cytokine IL-10 and adiponectin, which modulates several metabolic processes and has an anti-inflammatory effect [138], and a decrease in mitochondrial area with no changes in the number of mitochondria [134]. As documented in rat lung cells, ROS may also trigger mitophagy, a mechanism aimed at promoting cellular adaptation and reducing oxidative stress-induced damage through the elimination and recycling of damaged cells and organelles [139]. Mitophagy involves the activation of the PINK1/Parkin signaling pathway and the increased expression of protein LC-II, which, upon exposure to O_3_, are transferred to mitochondria where they prevent damaged mitochondrial fusion and induce ubiquitination and proteasomal degradation [139]. Furthermore, mitophagy can induce pyroptosis, a type of inflammatory programmed cell death through the activation of the NLRP3 inflammasome, a member of the cytoplasmic receptor family able to interact with oxidized mitochondrial DNA, which, in turn, activates caspase-1, which promotes the maturation and the consequent release of IL-1β and IL-18 by macrophages and dendritic cells [139,140].
3. Like other air pollutants, O_3_ appears to cause epigenetic alterations. Rats subjected to acute inhalation of O_3_ showed reduced levels of lung genes *Dnmt3a* and *Dnmt3b* isoforms, which are responsible for de novo methylation, while no significant changes were detected in the expression of *Dnmt1,* which exerts a maintenance action on DNA methylation [141]. In addition, overall pulmonary Dnmt activity was significantly reduced in response to O_3_ exposure [141]. Also, the expression of apelin, a hormone widely distributed in various tissue types both in humans and rodents but having the highest concentration in the placenta, lungs, and brain, was substantially and significantly reduced in rats exposed to O_3_ [141]. Importantly, the activation of the apelinergic system leads to beneficial effects to health, preventing oxidative stress and the consequent inflammation and protecting cells from endoplasmic reticulum-induced cell death, and increased levels of apelin have a cardioprotective role [142,143]. Furthermore, this study reports an increased percentage in methylation on the apelin gene in the promoter and primer regions in O_3_-exposed rats compared to controls [142]. Of note, a longitudinal study [144] conducted on 43 Chinese students aimed at measuring the effects of individual exposure to O_3_ on selected metabolites, showed that a 2 h increase in average O_3_ exposure was associated with higher levels of blood pressure, serum angiotensin-converting enzyme (ACE, a component of the hormonal renin–angiotensin system, which regulates blood pressure, [145]), and endothelin-1 (ET-1), which is a potent vasoconstrictor playing a pivotal role in maintaining vascular tone [146]. Additionally, acute exposure to O_3_ was also related to reduced methylation of *ACE* and *ET-1*, although in different degrees depending on the loci [144].

**Table 6:** Possible biological mechanisms underlying the relationship between exposure to carbon monoxide and ozone and the risk of congenital heart disease.

O_3_ Effects	Reference	CO_3_ Effects	Reference
Chronic exposure to O_3_ associated with reduced mitochondrial complex I, III, and V expression in lung bronchioles	[124]	Direct inhibition of CO on cytochrome C oxidase in muscle tissues	[127]
Direct oxidative action of O_3_ on proteins, lipids, and DNA	[122,125,126]	Increased blood CO concentration associated with reduced activity of cytochrome chain in brain	[129]
Induction of cytokine secretion and NF-κB in both lungs and cerebral cortex	[122,133]	Direct increase in ROS production from mitochondria in cortical neurons	[130]
Increased macrophage infiltration into epicardial adipose tissue	[134]	Induction of lipid peroxidation and decrease in glutathione levels in CNS cells	[129]
Upregulation of genes encoding TNF-α, monocyte chemoattractant protein-1, and leptin, and increase expression of NO synthase in epicardial and perirenal adipose tissues	[134]		
Downregulation of genes encoding IL-10 and adiponectin and decrease in mitochondrial area of epicardial and perirenal adipose tissues	[134]		
Acute inhalation of O_3_ associated with downregulation of Dnmt3a and Dnmt3b and of overall Dnmt activity in lungs	[141]		
O_3_-induced reduction in lung apelin expression associated with increased methylation levels in the apelin gene	[141]		
Acute exposure to O3 associated with higher levels of blood pressure	[144]		
Acute exposure to O3 associated with increased expression of serum ACE-2 and ET-1 and reduced methylation status of correspondent genes	[144]		

Abbreviations: ACE: angiotensin-converting enzyme; CO: carbon monoxide; CNS: central nervous system; Dnmt: DNA methyltransferase; ET-1: endothelin-1; NF-κB: nuclear factor kappa-light-chain-enhancer of activated B cells; NO: nitric oxide; O_3_: ozone; ROS: reactive oxygen species; TNF-α: tumor necrosis factor alpha.

## 3. Citizens and Environment: An Overview of Innovative Strategies for Pollution Monitoring and a Link for the Future in the Field of Air Pollution in CHD

**Figure 2:**
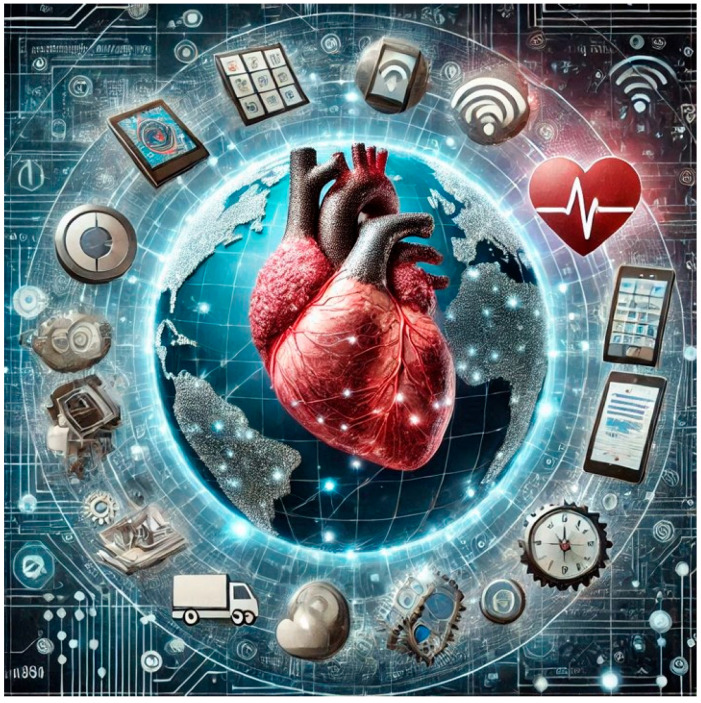
Paradigm of citizen science, using consumer technology and Artificial Intelligence to study the effects of air pollution on CHD (image partially generated by the Artificial Intelligence tool Microsoft Bing).

To further enhance the innovative contribution of this literature review, we also reviewed novel approaches and strategies to study the contribution of ambient air pollution to CHD occurrence. In this regard, the application of “hot topics” to the problem, including of the Artificial Intelligence (where relevant) and Internet-of-Things/Consumer Technologies, which are actually widespread across the global population, can represent a useful add-on to the current literature, providing, at a time, a significant link to future research activities in the domain (Figure 2). In such a framework, in recent years, thanks to the advent and rapid development of technological facilities, based on consumer technology, featuring low costs, ease of use and good interpretability, the concept of “citizen science” has come to attention of the scientific community and the wider public in general. In fact, citizen science refers to the collection and analysis of data relating to the natural world by members of the general public, typically as part of a collaborative project with professional scientists. In this regard, monitoring environmental pollutants represents one of the most popular use-case scenarios for citizen science, and one of the domains where the impact of technology has emerged as being among the most viable and reliable alternatives to classical monitoring methods.

### 3.1. Particulate Matter: A “Matter of Size”

PM monitoring devices are commonly used in everyday practice thanks to the good availability of commercial solutions merging together cost affordability and sufficient reliability. Portable monitors are currently available for a range of PM dust, like the Atmotube Pro (Atmotech Inc., San Francisco, CA, USA), capable of detecting particles up to 1 µg/m^3^ in size, or the Airthings View Plus (Airthings, Oslo, Norway), totally suitable for indoor environments, and ideal to capture PM_1_ and PM_2.5_ compounds. Small, portable, and even more affordable solutions are also available, like the Aeroqual Inc. (Auckland, New Zealand) PM_10_/PM_2.5_ Portable Particulate Monitor or the Prana Air Pocket PM_2.5_ (Purelogic Labs India, New Delhi, India); however, these are properly designed to detect the presence of bigger particles, typically 2.5 or 10 µM. A prototypal system, named Thingspeak, including an Internet of Things (IoT)-based platform, was used for monitoring, in real-time, data related to air quality, providing the user with an user-friendly digital dashboard on Smartphone/PC, in turn displaying air quality indicators, merging PM_1_ PM_2.5_, PM_10_, temperature, humidity, barometric pressure, Air Quality Index, dew point, and estimated altitude, sent to the cloud every 15 s [147]. Some interesting tools were developed for monitoring PM in specific sites, including construction sites and subway tunnels, where such compounds represent a real concern for the health of exposed individuals, including workers and citizens in general, respectively [148,149]. In the two cases, the results were overall satisfying, with questionable performances regarding accuracy only in the first scenario due to the specific principle of operation of the IoT detectors. However, in general, most studies report good detection performance, but also identify, as the main challenge for using portable technology to monitor PM, some uncertainties in PM measurements, the quality of data, and the related influence of environmental variables in the whole process [150].

### 3.2. Nitrogen Oxide and Sulfur Dioxide Monitoring: The Pathway to Artificial Intelligence

NO_2_ and SO_2_ are among the most popular pollutants commonly detected by major portable products available to date. Aeroqual Inc. (Auckland, New Zealand), already mentioned for its monitoring tools for PM, has developed the AQS 1 Air Quality Monitor, capable of providing an estimate of several particle size fractions, as well as a variety of gas pollutants, including NO_2_, SO_2_, O_3_, CO, hydrogen sulfide, methane, and VOC. NO_2_ is also captured by the Air Quality Meter Gasman-NO2 “Nitrogen Dioxide” solution (PCE Instruments, Manchester, UK) in a range between 0 and 10 ppm, with 1 ppm considered to be a threshold value for alarm according to the device, which is also capable of detecting SO_2_. The Sensible+ Air Monitor (Purelogic Labs India, New Delhi, India) merges a number of indicators to provide an overall Air Quality Index, combining them all in a single estimate and, at the same time, giving the user an exhaustive overview on the single pollutants, also including, among others, NO_2_ and SO_2_, with fairly good performance when compared with gold-standard measurement methods. Very interesting solutions with easy-to-use interfaces have been developed by Airsset (Vancouver, Canada), among which the Essential and the Nano devices are capable of detecting NO_2_ together with other gasses. Finally, SO_2_ was also captured with an IoT-based prototype developed in India, along with other pollutants [151], demonstrating the feasibility of this monitoring approach for this specific compound. An extremely affordable solution was developed in Morocco based on the Arduino hardware, including electronic devices, sensors, and wireless technology, with the ultimate aim of sensing the climate parameters, such as temperature, humidity, and concentration of some gasses, including SO_2_ and NO_2_ [152]. Interesting results were also obtained in a paper describing the implementation of a solution for monitoring pollutants like PM_2.5_ and PM_10_, O_3_, CO, NO_2_, and nitrogen trihydride by means of three sensors (PMSA003, MICS-6814, and MQ-131), being equipped with the ESP-WROOM-32 microcontroller featuring Wi-Fi and Bluetooth connection to send data to the cloud [153]. A significant step forward in this direction was made by Almalawi and colleagues [154], who combined the advancements of the IoT approach with those of Artificial Intelligence (AI) for data analysis. In particular, they developed three AI models for forecasting pollution levels and a predictive model for four gasses, namely CO_2_, SO_2_, NO_2_, and PM, differently, using Linear Regression, Support Vector Regression, and the Gradient Boosted Decision Tree GBDT Ensembles model.

### 3.3. Ozone and Carbon Monoxide: Portable Devices for Their Accurate Detection

Finally, O_3_ and CO are also frequently monitored by popular analyzers. The Series 500 Portable Ozone Monitor (Aeroqual Inc., Auckland, New Zealand) is a real-time ozone monitor, mounted within a portable device. It is suitable for O_3_ source and leak detection, process control, and health and safety monitoring. Also suitable for industrial monitoring, the PTM600OZ ozone gas analyzer (Beijing Zetron Technology Co., Ltd., Beijing, China) can detect the concentration of O_3_ according to UV light intensity changes. The portable dissolved ozone meter OZ-21P (DKK-TOA Corporation, Tokyo, Japan) is able to measure the water discharge concentration of O_3_ water generators without reagents in a concentration range of 0–2.00 mg/L and within a temperature between 5 and 40 °C. The HiYi LH-M900 Portable multi-parameter (Beijing Hiyi Technology Co., Ltd., Beijing, China) is a water quality analyzer for Ozone, Nitrite, Nitrate, Phosphate, and Oxygen, combining good performance and cost affordability. From Shenzhen Langdeli Technology Co., Ltd. (Shenzhen, China), a small, compact, user-friendly product, the Portable O_3_ Gas Detector Ozone Concentration Measurement Meter Handheld Air Pollution Tester Analyzer for PM_2.5_, and total VOC was developed, representing an important add-on to the solutions currently on the market, ideal for its cost affordability and ease of use.

As for CO, the TG-CO-828 (Tecnogas, Padua, Italy) is a portable digital detector, which is practical to install, easy to use, and esthetically attractive, capable of displaying, in real-time, the CO concentration in the air, and being suitable for a range of applications, including home monitoring, fireplace areas, and so forth. The First Alert Plug-In Carbon Monoxide Detector (First Alert, Aurora, IL, USA), equipped with electrochemical sensors, can detect CO from anywhere within a room or closed environment, making it ideal for home monitoring. Finally, another device worth mentioning is the Kidde carbon monoxide detector, featuring ease of use, a long-lasting battery, and a bulk alarm for CO threshold range overtaking.

In the research environment, a smart solution based on a low-power architecture featuring low-cost Raspberry Pi for data acquisition was developed in India, with fairly good results when it comes to the detection of several pollutants, including PM_2.5_, CO, carbon dioxide, temperature, humidity, and air pressure [155]. Finally, a very interesting work dealt with the detection of CO, monitored, also thanks to a recently deployed Wireless Sensor Network, using the Zigbee protocol for agile, low-cost, efficient communication [156].

## 4. Conclusions

**Figure 3:**
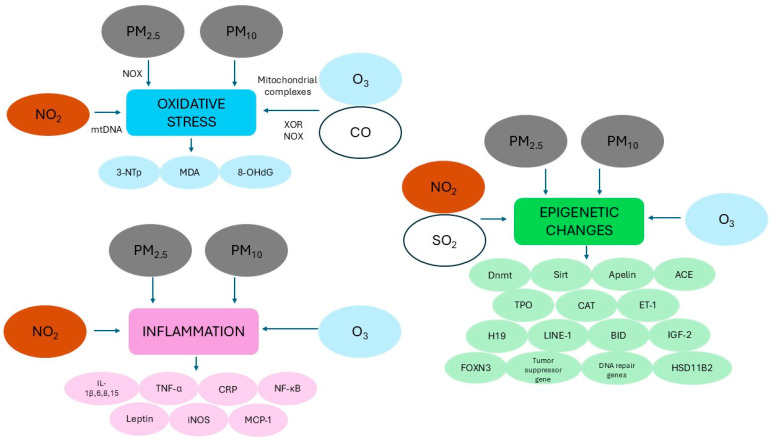
Schematic representation of the main mechanisms hypothesized to explain the potential role of ambient air pollutants that increase the risk of congenital heart disease (see text for more details). Abbreviations: 3-NTp: 3-nitrotyrosine; 8-OHdG: 8-hydroxy-2′-deoxyguanosine; ACE: angiotensin-converting enzyme; BID: BH3 interacting domain death agonist; CAT: catalase; CO: carbon monoxide; CRP: C-reactive protein; Dnmt: DNA methyltransferase; ET-1: endothelin 1; FOXN3: forkhead box N3; HSD11B2: 11β-hydroxysteroid dehydrogenase 2; IGF-2: insulin-like growth factor 2; IL: interleukin; iNOS: inducible nitric oxide synthase; LINE: long interspersed nucleotide element MCP-1: monocyte chemoattractant protein-1; MDA: malondialdehyde; mtDNA: mitochondrial DNA NO_2_: nitrogen dioxide; NF-κB: nuclear factor kappa-light-chain-enhancer of activated B cells; NOX: NADPH oxidase; O_3_: ozone; PM_2.5_: particulate matter with aerodynamic diameter OF less than 2.5 µM; PM_10_: particulate matter with aerodynamic diameter OF less than 10 µM; SO_2_: sulfur dioxide; Sirt: sirtuin; TNF-α: tumor necrosis factor alpha; TPO: thyroid peroxidase; XOR: xanthine oxidoreductase.

This review, which aims to comprehensively summarize the current knowledge on the relationship between maternal exposure to air pollution and the onset of CHD, which still represents the leading cause of birth-defect mortality, suggests the potential role of ambient air pollutants in increasing the risk of CHD, although with varying degrees of evidence. Air pollution is currently one of the greatest threats to human health, and reducing atmospheric emissions is among the major future challenges for world governments. While PM, and particularly PM_2.5_, shows moderate evidence of association with risk of CHD both during pregnancy and the preconceptional period with some signals of association with certain CHD subtypes (i.e., ASD, ToF, and TGA), for other pollutants, the data are generally inconsistent. Indeed, for NO_2_, SO_2_, and CO, the results are conflicting, with positive, inverse, or null associations with both overall CHD and CHD subtypes, while some evidence of association emerges in recent studies between exposure to O_3_ during the periconceptional period and increased occurrence of pooled CHD and several CHD subtypes. Although experimental studies and, to a lesser extent, human studies have identified a set of mechanisms, namely oxidative stress and related inflammation, and epigenetics, which may explain the biological plausibility of the involvement of these pollutants in the development of CHD (Figure 3), numerous issues need to be addressed before definitive conclusion can be drawn.

In fact, most of the studies conducted so far were conducted in China, notoriously one of the most polluted countries at the global level, and this could have produced association estimates that should be verified in world regions characterized by a lower degree of air pollution. In addition, the published studies present a misclassification of exposure and are characterized by a high heterogeneity in exposure windows (preconception, periconception, and early-, mid-, or late-pregnancy), pollutant concentrations, target populations (with a predominance of live-births), and confounding factors, which make association estimates imprecise and difficult to compare. Therefore, future cohort studies, in addition to being performed in countries with different levels of air pollution and using standardized and advanced methods of exposure assessments such as models based on multiple data sources that can accurately estimate individual exposure, should include both data on fetal losses, still births, and terminations of pregnancy and a wide range of further risk factors in order to reduce the risk of outcome bias. At the same time, molecular studies on animal models and humans could help provide crucial information to determine a comprehensive risk assessment. Given the great impact of CHD on individuals and public health, a deep understanding of CHD etiology would be essential to timely and effectively manage modifiable risk factors, such as air pollutants, possibly mitigating the exposure of future mothers, especially during the periconceptional period and the first weeks of gestation, to reduce the risk of developing CHD and improve disease prognosis. However, given the overall uncertainty about the gestational windows most sensitive to the effects of air pollution, future epidemiological studies should evaluate the association between exposure at different stages of pregnancy, also considering multiple exposures and the risk of CHD. Greater attention should be paid to preventing exposure of pregnant women living in highly polluted areas, where the main sources of pollution are from high-density industries and traffic vehicles. In this regard, citizens awareness and self-monitoring of environmental conditions, performed thanks to new technologies, mainly referring to IoT and AI for subsequent data analysis, can represent new insights supporting a larger amount of data to be collected and analyzed and overtake current methodological limitations such as, for example, the availability of data which are not necessarily representative of the real exposure of a family or small group of individuals and thus drive toward spurious evidence. Finally, given the great differences in access to prenatal diagnosis between world regions, primary prevention and information programs on the risk of air pollution exposure should be implemented in low-income countries, which sometimes coincide with the highest pollution rates.

## Figures and Tables

**Table 1:** Clues and pitfalls in the relationship between prenatal exposure to particulate matter and congenital heart disease.

Clues	Reference	Pitfalls	Reference
Significant association between PM_2.5_ exposure during pregnancy and increased risk of CHD	[46,58]	No significant association between maternal PM_2.5_ exposure and risk of overall CHD and CHD subtypes	[15]
Significantly positive association between periconceptional PM_2.5_ exposure, particularly during the preconceptional period, to PM_2.5_ and risk of overall CHD and septal defects	[7]	No information on mother’s mobility during pregnancy	[46,58]
Significant association between continuous PM_2.5_ exposure and TGA occurrence	[15]	No data on the distance between air quality monitoring stations and residential/work address of mothers	[46,58]
Categorical and continuous PM_10_ exposure during pregnancy significantly associated with increased risk of overall CHD	[15,59]	No pregnancy terminations before the 28 weeks of gestation included in the exposure assessment	[7,46]
Prenatal exposure to PM_2.5_ significantly associated with increased occurrence of ToF	[58]	Neonates beyond 42 days after birth considered as controls	[7]
Significant association of prenatal PM_10_ exposure with risk of ASD.	[59]	Possible residual confounding factors (time spent outdoors, time spent at work, genetic features of parents and newborns, passive smoking, maternal education, folate supplementation, and alcohol consumption) not included	[7,46,58]
		PM_2.5_ showing a wide exposure range and with limited sample size at high concentrations	[7]
		Possibility of recall bias in case–control studies	[7,46,58]
		Lack of assessment of ethnic minority risk factors and different ethnic groups.	[58]
		Heterogeneity between studies and systematic reviews due to differences in measurement methods of exposure, exposure window, study design, target populations, concentration of pollutants, different diagnostic systems, outcome definitions, and confounding factors	[15,59]
		Possibility of publication bias	[15,59]
		Multiple potential sources of bias	[59]
		High statistical heterogeneity of effect estimates	[59]
		No possibility of causal inferences due to the case–control design	[7,46,58]

Abbreviations: ASD: atrial septal defects; CHD: congenital heart disease; PM_2.5_: particulate matter with aerodynamic diameter of less than 2.5 µM; PM_10_: particulate matter with aerodynamic diameter of less than 10 µM; TGA: transposition of great arteries; ToF: tetralogy of Fallot.

**Table 2:** Possible biological mechanisms underlying the relationship between exposure to particulate matter and the risk of congenital heart disease.

Effects	Reference
Promotion of oxidative stress through endogenous overproduction of ROS and increased expression of Nox4 in the heart	[60,62,63,64,65,66]
Positive association between PM_2.5_ exposure throughout pregnancy and placental levels of 3-NTp	[65]
Positive association between PM_10_ exposure during the first and the second trimester of gestation and mitochondrial levels of 8-OHdG	[67]
Positive association between cumulative PM_2.5_ exposure during the second trimester of gestation and urinary 8-OHdG levels	[68]
Positive association between PM_2.5_ exposure during the first trimester of gestation and urinary MDA levels	[68]
In utero exposure to PM_2.5_ associated with decreased expression of myocardial Sirt1 and Sirt2 and increased expression of Dnmt1, Dnmt3a, and Dnmt3b	[70]
Preconceptional exposure to PM_2.5_ associated with increased expression of myocardial Sir1 and Sirt2 and reduced expression of Dnmt1, with no changes in Dnmt3a	[71]
Positive association between PM_2.5_ exposure during the second trimester of gestation and methylation status of leptin in placental trophoblasts	[72]
Significant association between prenatal exposure to PM_2.5_ and epigenetic changes in placental *APEX1*, *OGG*, *ERCC4*, *p53*, and *DAPK1* and *BID*, *IGF2*, and *FOXN3*	[69,74]
Significant association between prenatal exposure to PM_2.5_ and epigenetic changes in placental *BID* and *IGF2* (entire pregnancy) and *FOXN3* (second trimester)	[69]
Exposure to PM_10_ significantly associated with placental decreased methylation of *LINE-1* (first trimester) and increased methylation of *HSD11B2* (first and second trimesters) in the placenta	[82]
Positive association between PM_10_ exposure and *H19* DMR methylation in cord blood (entire pregnancy) and maternal blood (third trimester)	[83]
Significantly negative associations between gestational exposure to PM_1_, PM_2.5_, and PM_10_ and cord blood LINE-1 methylation levels and between PM_1_ exposure and maternal LINE-1 methylation levels	[85]
Prenatal and gestational exposure to PM_2.5_ positively associated with myocardial levels of IL-1β, IL-6, IL-8, IL-15, TNF-α, CRP, E-selectin, and P-selectin	[66,72,87]
High levels of exposure to PM_10_ positively associated with levels of maternal blood CRP (during early pregnancy) and fetal cord blood CRP (during the entire pregnancy)	[88]

Abbreviations: 3-NTp: 3-nitrotyrosine; 8-OHdG: 8-hydroxy-2′-deoxyguanosine; CRP: C reactive protein; Dnmt: DNA methyltransferase; IL-interleukin; LINE: long interspersed nucleotide element; Nox4: NADPH oxidase 4; PM_1_: particulate matter with aerodynamic diameter of less than 1 µM PM_2.5_: particulate matter with aerodynamic diameter of less than 2.5 µM; PM_10_: particulate matter with aerodynamic diameter of less than 10 µM;.ROS: reactive oxygen species; Sirt: sirtuin; TNF-α: tumor necrosis factor alpha.

**Table 3:** Clues and pitfalls in the relationship between prenatal exposure to nitrogen and sulfur dioxide and congenital heart disease.

Clues	Reference	Pitfalls	Reference
Increased risk of CoA in relation to prenatal exposure to NO_2_	[59]	Null or significantly inverse association between maternal SO_2_ exposure and risk of overall CHD, VSD, and ToF	[15,46]
Possibility of positive association between maternal NO_2_ exposure and risk of CHD and ToF	[46,58,59]	Lack of significant association between NO_2_ exposure during pregnancy and risk of overall CHD or CHD subtypes	[15,59]
Marked positive association between SO_2_ exposure during the first and second months of gestation and CHD occurrence	[58]	No significant associations of NO_2_ and SO_2_ exposure in the first and second months of pregnancy with the occurrence of CHD in a multi-pollutant model	[58]
Possibility of association of prenatal exposure to SO_2_ and risk of CoA and ToF in offspring	[59]	No information on mother’s mobility during pregnancy	[46,58]
		No data on the distance between air quality monitoring stations and residential/work address of mothers	[46,58]
		No pregnancy terminations before 28 weeks of gestation included in the exposure assessment	[46]
		Possible residual confounding factors (time spent outdoors, time spent at work, genetic features of parents and newborns, passive smoking, maternal education, folate supplementation, and alcohol consumption) not included	[46,58]
		Lack of assessment of ethnic minority risk factors and different ethnic groups	[58]
		Heterogeneity between both studies and systematic reviews due to differences in measurement methods of exposure, exposure window, study design, target populations, concentration of pollutants, different diagnostic systems, outcome definitions, and confounding factors	[15,59]
		Possibility of publication bias and recall bias	[15,46,58,59]
		Multiple potential sources of bias	[59]
		High statistical heterogeneity of effect estimates	[59]
		No possibility of causal inferences due to the case–control design	[46,58]

Abbreviations: ASD: atrial septal defects; CHD: congenital heart disease; CoA: coarctation of aorta; NO_2_: nitrogen dioxide; SO_2_: sulfur dioxide; ToF: tetralogy of Fallot; VSD: ventricular septal defects.

**Table 5:** Clues and pitfalls in the relationship between prenatal exposure to ozone and carbon monoxide and congenital heart disease.

Clues	Reference	Pitfalls	Reference
Significantly positive association of maternal O_3_ exposure in the preconceptional, periconceptional, and early pregnancy periods with occurrence of CHD	[58,113,121]	Borderline positive or negative association of maternal CO exposure with CHD risk	[46,58]
Marked increased risk of ToF related to gestational exposure to CO in both continuous and categorical exposures	[15]	No significant association found between O_3_ exposure during pregnancy and risk of CHD	[15]
Significantly positive associations between maternal exposure to O_3_ in the first 3–8 weeks of pregnancy and increased risk of VSD, ToF, PA, PS, TGA, and PLSVC	[121]	No information on mother’s mobility during pregnancy	[46,58,113]
Significantly positive associations between maternal exposure to O_3_ in the periconceptional period and increased risk of VSD, VR, ToF, PA, PS, TGA, and PLSVC	[121]	No data on the distance between air quality monitoring stations and residential/work address of mothers	[46,58]
		No pregnancy terminations before the 28 weeks of gestation included in the exposure assessment	[46]
		Possible residual confounding factors (time spent outdoors, time spent at work, genetic features of parents and newborns, diet, physical activity, passive smoking, maternal education, folate supplementation, alcohol consumption, use of air conditioner, socioeconomic status, and pre-pregnancy BMI) not included	[46,58,113,121]
		Exposure misclassification can underestimate or overestimate the observed effects	[113,121]
		Lack of assessment of ethnic minority risk factors and different ethnic groups or from a single province	[58,121]
		Heterogeneity between studies and systematic reviews due to differences in measurement methods of exposure, exposure window, study design, target populations, concentration of pollutants, different diagnostic systems, outcome definitions, and confounding factors	[15,59]
		Possibility of publication bias and recall bias	[15,46,58,59,113,121]
		Multiple potential sources of bias	[59]
		High statistical heterogeneity of effect estimates	[59]
		No possibility of causal inferences due to the case–control design	[46,58,113,121]

Abbreviations: ASD: atrial septal defects; BMI: body mass index; CHD: congenital heart disease; CO.: carbon monoxide; O_3_: ozone; PA: pulmonary atresia; PLSVC: persistent left superior vena cava; PS: pulmonary stenosis; TGA: transposition of great arteries; ToF: tetralogy of Fallot; VR: vascular ring of aorta; VSD: ventricular septal defects.

## Abbreviations

3-NTp	Ken-ichiro Tanaka3-nitrotyrosine
8-OHdG	8-hydroxy-2′-deoxyguanosine
AI	Artificial Intelligence
ASD	Atrial septal defects
CHD	Congenital heart disease
CO	Carbon monoxide
CoA	Coarctation of aorta
CRP	C-reactive protein
Dnmt	DNA methyltransferase
IQR	Interquartile range
IL	Interleukin
IoT	Internet of Things
LBW	Low birth weight
mtDNA	Mitochondrial DNA
NF-κB	Nuclear factor kappa-light-chain-enhancer of activated B cells
NO	Nitrogen dioxide
NOX	NADPH oxidase
O_3_	Ozone
PDA	Patent ductus arteriosus
PM	Particulate matter
PTB	Preterm birth
ROS	Reactive oxygen species
SGA	Small per gestational age
Sirt	Sirtuin
SO_2_	Sulfur dioxide
TGA	Transposition of great arteries
TNF-α	Tumor necrosis factor alpha
ToF	Tetralogy of Fallot
VOC	Volatile organic compounds
VSD	Ventricular septal defects

## References

[1] Yao Z., Xie W., Zhang J., Yuan H., Huang M., Shi Y., Xu X., Zhuang J. (2023). Graph matching and deep neural networks based whole heart and great vessel segmentation in congenital heart disease. Sci. Rep..

[2] Abdul-Khaliq H., Gomes D., Meyer S., von Kries R., Wagenpfeil S., Pfeifer J., Poryo M. (2024). Trends of mortality rate in patients with congenital heart defects in Germany-analysis of nationwide data of the Federal Statistical Office of Germany. Clin. Res. Cardiol..

[3] Su Z., Zou Z., Hay S.I., Liu Y., Li S., Chen H., Naghavi M., Zimmerman M.S., Martin G.R., Wilner L.B. (2022). Global, regional, and national time trends in mortality for congenital heart disease, 1990–2019: An age-period-cohort analysis for the Global Burden of Disease 2019 study. EClinicalMedicine.

[4] Liu Y., Chen S., Zühlke L., Black G.C., Choy M.K., Li N., Keavney B.D. (2019). Global birth prevalence of congenital heart defects 1970–2017: Updated systematic review and meta-analysis of 260 studies. Int. J. Epidemiol..

[5] Hasan A.A., Abu Lehyah N.A.A., Al Tarawneh M.K., Abbad M.Y., Fraijat A.G., Al-Jammal R.A., Moamar D.M., Shersheer Q.A., Guthrie S.O., Starnes J.R. (2023). Incidence and types of congenital heart disease at a referral hospital in Jordan: Retrospective study from a tertiary center. Front. Pediatr..

[6] Roth G.A., Mensah G.A., Johnson C.O., Addolorato G., Ammirati E., Baddour L.M., Barengo N.C., Beaton A.Z., Benjamin E.J., Benziger C.P. (2020). Global Burden of Cardiovascular Diseases and Risk Factors, 1990–2019: Update From the GBD 2019 Study. J. Am. Coll. Cardiol..

[7] Yuan X., Liang F., Zhu J., Huang K., Dai L., Li X., Wang Y., Li Q., Lu X., Huang J. (2023). Maternal Exposure to PM2.5 and the Risk of Congenital Heart Defects in 1.4 Million Births: A Nationwide Surveillance-Based Study. Circulation..

[8] Baldacci S., Gorini F., Minichilli F., Pierini A., Santoro M., Bianchi F. (2016). Rassegna degli studi epidemiologici su fattori di rischio individuali e ambientali nell’eziologia dei difetti cardiaci congeniti [Review of epidemiological studies on individual and environmental risk factors in the aetiology of congenital heart defects]. Epidemiol. Prev..

[9] Rachamadugu S.I., Miller K.A., Lee I.H., Zou Y.S. (2022). Genetic detection of congenital heart disease. Gynecol. Obstet. Clin. Med..

[10] Yang J., Kang Y., Cheng Y., Zeng L., Yan H., Dang S. (2019). Maternal Dietary Patterns during Pregnancy and Congenital Heart Defects: A Case-Control Study. Int. J. Environ. Res. Public Health.

[11] Turunen R., Pulakka A., Metsälä J., Vahlberg T., Ojala T., Gissler M., Kajantie E., Helle E. (2024). Maternal Diabetes and Overweight and Congenital Heart Defects in Offspring. JAMA Netw. Open.

[12] Bolin E.H., Gokun Y., Romitti P.A., Tinker S.C., Summers A.D., Roberson P.K., Hobbs C.A., Malik S., Botto L.D., Nembhard W.N. (2022). Maternal Smoking and Congenital Heart Defects, National Birth Defects Prevention Study, 1997–2011. J. Pediatr..

[13] Zhang T.N., Wu Q.J., Liu Y.S., Lv J.L., Sun H., Chang Q., Liu C.F., Zhao Y.H. (2021). Environmental Risk Factors and Congenital Heart Disease: An Umbrella Review of 165 Systematic Reviews and Meta-Analyses With More Than 120 Million Participants. Front. Cardiovasc. Med..

[14] Gorini F., Tonacci A. (2023). Toxic metals in pregnancy and congenital heart defects. Insights and new perspectives for a technology-driven reduction in food sources. Explor. Cardiol..

[15] Wan X., Wei S., Wang Y., Jiang J., Lian X., Zou Z., Li J. (2023). The association between maternal air pollution exposure and the incidence of congenital heart diseases in children: A systematic review and meta-analysis. Sci. Total Environ..

[16] Mullen M., Zhang A., Lui G.K., Romfh A.W., Rhee J.W., Wu J.C. (2021). Race and Genetics in Congenital Heart Disease: Application of iPSCs, Omics, and Machine Learning Technologies. Front. Cardiovasc. Med..

[17] WHO, World Health Organization (2024). Ambient (Outdoor) Air Pollution. https://www.who.int/newsroom/fact-sheets/detail/ambient-(outdoor)-air-quality-and-health.

[18] Isola S., Murdaca G., Brunetto S., Zumbo E., Tonacci A., Gangemi S. (2024). The Use of Artificial Intelligence to Analyze the Exposome in the Development of Chronic Diseases: A Review of the Current Literature. Informatics.

[19] Klepac P., Locatelli I., Korošec S., Künzli N., Kukec A. (2018). Ambient air pollution and pregnancy outcomes: A comprehensive review and identification of environmental public health challenges. Environ. Res..

[20] Mitku A.A., Zewotir T., North D., Jeena P., Asharam K., Muttoo S., Tularam H., Naidoo R.N. (2023). Impact of ambient air pollution exposure during pregnancy on adverse birth outcomes: Generalized structural equation modeling approach. BMC Public Health.

[21] Ravindra K., Chanana N., Mor S. (2021). Exposure to air pollutants and risk of congenital anomalies: A systematic review and metaanalysis. Sci. Total Environ..

[22] Yang B.Y., Qu Y., Guo Y., Markevych I., Heinrich J., Bloom M.S., Bai Z., Knibbs L.C., Li S., Chen G. (2021). Maternal exposure to ambient air pollution and congenital heart defects in China. Environ. Int..

[23] WHO, World Health Organization (2024). Air Quality, Energy and Health. https://www.who.int/teams/environment-climate-change-and-health/air-quality-energy-and-health/health-impacts/exposure-air-pollution#:~:text=An%20estimated%204.2%20million%20deaths,cancer%20and%20acute%20respiratory%20infections.

[24] Euroepean Environment Agency (2023). World Health Organization (WHO) Air Quality Guidelines (AQGs) and Estimated Reference Levels (RLs). https://www.eea.europa.eu/publications/status-of-air-quality-in-Europe-2022/europes-air-quality-status-2022/world-health-organization-who-air.

[25] European Environment Agency (2022). Air Quality in Europe 2022. https://www.eea.europa.eu/publications/air-quality-in-europe-2022/.

[26] WHO, World Health Organization (2024). Air Pollution. https://www.who.int/health-topics/air-pollution#tab=tab_1.

[27] US EPA, United States Environmental Protection Agency (2024). Outdoor Air Quality. https://www.epa.gov/report-environment/outdoor-air-quality.

[28] European Environment Agency (2016). Dispersal of Air Pollutants. https://www.eea.europa.eu/publications/2599XXX/page005.html.

[29] Kortoçi P., D Motlagh N.H., Zaidan M.A., Fung P.L., Varjonen S., Rebeiro-Hargrave A., Niemi J.V., Nurmi P., Hussein T., Petäjä T. (2021). Air pollution exposure monitoring using portable low-cost air quality sensors. Smart Health.

[30] Anedda M., Fadda M., Girau R., Pau G., Giusto D. (2023). A social smart city for public and private mobility: A real case study. Comput. Netw..

[31] Jebasingh F., Thomas N. (2022). Barker Hypothesis and Hypertension. Front. Public Health.

[32] Edwards M., Preedy V.R., Patel V.B. (2017). The Barker Hypothesis. Handbook of Famine, Starvation, and Nutrient Deprivation.

[33] Melody S., Wills K., Knibbs L.D., Ford J., Venn A., Johnston F. (2020). Adverse birth outcomes in Victoria, Australia in association with maternal exposure to low levels of ambient air pollution. Environ. Res..

[34] Luyten L.J., Saenen N.D., Janssen B.G., Vrijens K., Plusquin M., Roles H.A., Debacq-Chainiaux F., Nawrot T.S. (2018). Air pollution and the fetal origin of disease: A systematic review of the molecular signatures of air pollution exposure in human placenta. Environ. Res..

[35] Ghazi T., Naidoo P., Naidoo R.N., Chuturgoon A.A. (2021). Prenatal Air Pollution Exposure and Placental DNA Methylation Changes: Implications on Fetal Development and Future Disease Susceptibility. Cells.

[36] van den Hooven E.H., Pierik F.H., de Kluizenaar Y., Hofman A., van Ratingen S.W., Zandveld P.Y., Russcher H., Lindemans J., Miedema H.M., Steegers E.A. (2012). Air pollution exposure and markers of placental growth and function: The generation R study. Environ. Health Perspect..

[37] Zhu X., Liu Y., Chen Y., Yao C., Che Z., Cao J. (2015). Maternal exposure to fine particulate matter (PM2.5) and pregnancy outcomes: A meta-analysis. Environ. Sci. Pollut. Res. Int..

[38] Yuan L., Zhang Y., Gao Y., Tian Y. (2019). Maternal fine particulate matter (PM2.5) exposure and adverse birth outcomes: An updated systematic review based on cohort studies. Environ. Sci. Pollut. Res. Int..

[39] Zhou W., Ming X., Yang Y., Hu Y., He Z., Chen H., Li Y., Cheng J., Zhou X. (2023). Associations between maternal exposure to ambient air pollution and very low birth weight: A birth cohort study in Chongqing, China. Front. Public Health.

[40] Wang X., Wang X., Gao C., Xu X., Li L., Liu Y., Li Z., Xia Y., Fang X. (2023). Relationship Between Outdoor Air Pollutant Exposure and Premature Delivery in China- Systematic Review and Meta-Analysis. Int. J. Public Health.

[41] Cheng Y., Yin J., Yang L., Xu M., Lu X., Huang W., Dai G., Sun G. (2023). Ambient air pollutants in the first trimester of pregnancy and birth defects: An observational study. BMJ Open.

[42] Dadvand P., Rankin J., Rushton S., Pless-Mulloli T. (2011). Ambient air pollution and congenital heart disease: A register-based study. Environ. Res..

[43] Agay-Shay K., Friger M., Linn S., Peled A., Amitai Y., Peretz C. (2013). Air pollution and congenital heart defects. Environ. Res..

[44] Tanner J.P., Salemi J.L., Stuart A.L., Yu H., Jordan M.M., DuClos C., Cavicchia P., Correia J.A., Watkins S.M., Kirby R.S. (2015). Associations between exposure to ambient benzene and PM(2.5) during pregnancy and the risk of selected birth defects in offspring. Environ. Res..

[45] Tan C.M.J., Lewandowski A.J. (2020). The Transitional Heart: From Early Embryonic and Fetal Development to Neonatal Life. Fetal Diagn. Ther..

[46] Sun L., Wu Q., Wang H., Liu J., Shao Y., Xu R., Gong T., Peng X., Zhang B. (2023). Maternal exposure to ambient air pollution and risk of congenital heart defects in Suzhou, China. Front. Public Health.

[47] US EPA, United States Environmental Protection Agency (2024). Particulate Matter (PM) Basics. https://www.epa.gov/pm-pollution/particulate-matter-pm-basics.

[48] Thangavel P., Park D., Lee Y.C. (2022). Recent Insights into Particulate Matter (PM2.5)-Mediated Toxicity in Humans: An Overview. Int. J. Environ. Res. Public Health.

[49] US EPA, United States Environmental Protection Agency (2024). Particle Pollution Exposure. https://www.epa.gov/pmcourse/particle-pollution-exposure.

[50] Xie W., You J., Zhi C., Li L. (2021). The toxicity of ambient fine particulate matter (PM2.5) to vascular endothelial cells. J. Appl. Toxicol..

[51] Gangwar R.S., Bevan G.H., Palanivel R., Das L., Rajagopalan S. (2020). Oxidative stress pathways of air pollution mediated toxicity: Recent insights. Redox Biol..

[52] McDuffie (2021). E.E.; Martin, R.V.; Spadaro, J.V.; Burnett, R.; Smith, S.J.; O’Rourke, P.; Hammer, M.S.; van Donkelaar, A.; Bindle, L.; Shah, V.; Jaeglé, L.; et al. Source sector and fuel contributions to ambient PM2.5 and attributable mortality across multiple spatial scales. Nat. Commun..

[53] Rattanapotanan T., Thongyen T., Bualert S., Choomanee P., Suwattiga P., Rungrattanaubon T., Utavong T., Phupijit J., Changplaiy N. (2023). Secondary sources of PM2.5 based on the vertical distribution of organic carbon, elemental carbon, and water-soluble ions in Bangkok. Environ. Adv..

[54] Sang S., Chu C., Zhang T., Chen H., Yang X. (2022). The global burden of disease attributable to ambient fine particulate matter in 204 countries and territories, 1990–2019: A systematic analysis of the Global Burden of Disease Study 2019. Ecotoxicol Environ Saf..

[55] WHO, World Health Organization (2021). What are the WHO Air quality guidelines?. https://www.who.int/news-room/feature-stories/detail/what-are-the-who-air-quality-guidelines.

[56] WHO, World Health Organization (2021). WHO Global Air Quality Guidelines. Particulate Matter (PM2.5 and PM10), Ozone, Nitrogen Dioxide, Sulfur Dioxide and Carbon Monoxide.

[57] European Environment Agency (2024). Europe’s Air Quality Status 2024. https://www.eea.europa.eu/publications/europes-air-quality-status-2024.

[58] Huang Z., Qiu Y., Qi J., Ma X., Cheng Q., Wu J. (2023). Association between air pollutants and birth defects in Xiamen, China. Front. Pediatr..

[59] Michel S., Atmakuri A., von Ehrenstein O.S. (2023). Prenatal exposure to ambient air pollutants and congenital heart defects: An umbrella review. Environ. Int..

[60] Kunovac A., Hathaway Q.A., Pinti M.V., Taylor A.D., Hollander J.M. (2020). Cardiovascular adaptations to particle inhalation exposure: Molecular mechanisms of the toxicology. Am. J. Physiol. Heart Circ. Physiol..

[61] Saenen N.D., Martens D.S., Neven K.Y., Alfano R., Bové H., Janssen B.G., Roles H.A., Plusquin M., Vrijens K., Nawrot T.S. (2019). Air pollution-induced placental alterations: An interplay of oxidative stress, epigenetics, and the aging phenotype?. Clin. Epigenetics.

[62] Miller M.R., Shaw C.A., Langrish J.P. (2012). From particles to patients: Oxidative stress and the cardiovascular effects of air pollution. Future Cardiol..

[63] Miller M.R. (2020). Oxidative stress and the cardiovascular effects of air pollution. Free Radic. Biol. Med..

[64] Leni Z., Künzi L., Geiser M. (2020). Air pollution causing oxidative stress. Curr. Opin. Toxicol..

[65] Saenen N.D., Vrijens K., Janssen B.G., Madhloum N., Peusens M., Gyselaers W., Vanpoucke C., Lefebvre W., Roles H.A., Nawrot T.S. (2016). Placental Nitrosative Stress and Exposure to Ambient Air Pollution During Gestation: A Population Study. Am. J. Epidemiol..

[66] Wu F., Zhang J. (2018). The involvement of Nox4 in fine particulate matter exposure-induced cardiac injury in mice. J. Toxicol. Sci..

[67] Grevendonk L., Janssen B.G., Vanpoucke C., Lefebvre W., Hoxha M., Bollati V., Nawrot T.S. (2016). Mitochondrial oxidative DNA damage and exposure to particulate air pollution in mother-newborn pairs. Environ. Health.

[68] Wang X., Lin Y., Ge Y., Craig E., Liu X., Miller R.K., Thurston S.W., Brunner J., Barrett E.S., O’Connor T.G. (2024). Systemic oxidative stress levels during the course of pregnancy: Associations with exposure to air pollutants. Environ. Pollut..

[69] Zhao Y., Wang P., Zhou Y., Xia B., Zhu Q., Ge W., Li J., Shi H., Xiao X., Zhang Y. (2021). Prenatal fine particulate matter exposure, placental DNA methylation changes, and fetal growth. Environ. Int..

[70] Tanwar V., Gorr M.W., Velten M., Eichenseer C.M., Long V.P., Bonilla I.M., Shettigar V., Ziolo M.T., Davis J.P., Baine S.H. (2017). In Utero Particulate Matter Exposure Produces Heart Failure, Electrical Remodeling, and Epigenetic Changes at Adulthood. J. Am. Heart Assoc..

[71] Tanwar V., Adelstein J.M., Grimmer J.A., Youtz D.J., Katapadi A., Sugar B.P., Falvo M.J., Baer L.A., Stanford K.I., Wold L.E. (2018). Preconception Exposure to Fine Particulate Matter Leads to Cardiac Dysfunction in Adult Male Offspring. J. Am. Heart Assoc..

[72] Saenen N.D., Vrijens K., Janssen B.G., Roles H.A., Neven K.Y., Vanden Berghe W., Gyselaers W., Vanpoucke C., Lefebvre W., De Boever P. (2017). Lower Placental Leptin Promoter Methylation in Association with Fine Particulate Matter Air Pollution during Pregnancy and Placental Nitrosative Stress at Birth in the ENVIRONAGE Cohort. Environ. Health Perspect..

[73] Sagawa N., Yura S., Itoh H., Kakui K., Takemura M., Nuamah M.A., Ogawa Y., Masuzaki H., Nakao K., Fujii S. (2002). Possible role of placental leptin in pregnancy: A review. Endocrine..

[74] Neven K.Y., Saenen N.D., Tarantini L., Janssen B.G., Lefebvre W., Vanpoucke C., Bollati V., Nawrot T.S. (2018). Placental promoter methylation of DNA repair genes and prenatal exposure to particulate air pollution: An ENVIRONAGE cohort study. Lancet Planet. Health.

[75] Hu Z., Ding X., Ji Y., Liu X., Ding Z. (2021). APEX1 protects against oxidative damage-induced cardiomyocyte apoptosis. Biocell.

[76] Li W., Yu W., Xu W., Xiong J., Zhong X., Hu S., Yu J. (2021). Death-Associated Protein Kinase 1 Regulates Oxidative Stress in Cardiac Ischemia Reperfusion Injury. Cells Tissues Organs.

[77] Yan L., Zhou Y., Yu S., Ji G., Wang L., Liu W., Gu A. (2013). 8-Oxoguanine DNA glycosylase 1 (ogg1) maintains the function of cardiac progenitor cells during heart formation in zebrafish. Exp. Cell Res..

[78] Shen H., Gan P., Wang K., Darehzereshki A., Wang K., Kumar S.R., Lien C.L., Patterson M., Tao G., Sucov H.M. (2020). Mononuclear diploid cardiomyocytes support neonatal mouse heart regeneration in response to paracrine IGF2 signaling. Elife..

[79] Samaan G., Yugo D., Rajagopalan S., Wall J., Donnell R., Goldowitz D., Gopalakrishnan R., Venkatachalam S. (2010). Foxn3 is essential for craniofacial development in mice and a putative candidate involved in human congenital craniofacial defects. Biochem. Biophys. Res. Commun..

[80] Naumann B., Schmidt J., Olsson L. (2019). FoxN3 is necessary for the development of the interatrial septum, the ventricular trabeculae and the muscles at the head/trunk interface in the African clawed frog, *Xenopus laevis* (Lissamphibia: Anura: Pipidae). Dev. Dyn..

[81] Del Re D.P., Amgalan D., Linkermann A., Liu Q., Kitsis R.N. (2019). Fundamental Mechanisms of Regulated Cell Death and Implications for Heart Disease. Physiol. Rev..

[82] Cai J., Zhao Y., Liu P., Xia B., Zhu Q., Wang X., Song Q., Kan H., Zhang Y. (2017). Exposure to particulate air pollution during early pregnancy is associated with placental DNA methylation. Sci. Total Environ..

[83] He T., Zhu J., Wang J., Ren X., Cheng G., Liu X., Ma Q., Zhang Y., Li Z., Ba Y. (2018). Ambient air pollution, H19/DMR methylation in cord blood and newborn size: A pilot study in Zhengzhou City, China. Chemosphere..

[84] Miyaso H., Sakurai K., Takase S., Eguchi A., Watanabe M., Fukuoka H., Mori C. (2017). The methylation levels of the H19 differentially methylated region in human umbilical cords reflect newborn parameters and changes by maternal environmental factors during early pregnancy. Environ. Res..

[85] Liu X., Ye Y., Chen Y., Li X., Feng B., Cao G., Xiao J., Zeng W., Li X., Sun J. (2019). Effects of prenatal exposure to air particulate matter on the risk of preterm birth and roles of maternal and cord blood LINE-1 methylation: A birth cohort study in Guangzhou, China. Environ. Int..

[86] Crow M.K. (2010). Long interspersed nuclear elements (LINE-1): Potential triggers of systemic autoimmune disease. Autoimmunity..

[87] Zhao T., Qi W., Yang P., Yang L., Shi Y., Zhou L., Ye L. (2021). Mechanisms of cardiovascular toxicity induced by PM2.5: A review. Environ. Sci. Pollut. Res. Int..

[88] Craig R., Larkin A., Mingo A.M., Thuerauf D.J., Andrews C., McDonough P.M., Glembotski C.C. (2000). p38 MAPK and NF-kappa B collaborate to induce interleukin-6 gene expression and release. Evidence for a cytoprotective autocrine signaling pathway in a cardiac myocyte model system. J. Biol. Chem..

[89] van den Hooven E.H., de Kluizenaar Y., Pierik F.H., Hofman A., van Ratingen S.W., Zandveld P.Y., Lindemans J., Russcher H., Steegers E.A., Miedema H.M. (2012). Chronic air pollution exposure during pregnancy and maternal and fetal C-reactive protein levels: The Generation R Study. Environ. Health Perspect..

[90] US EPA, United States Environmental Protection Agency (2024). Basic Information about NO_2_. https://www.epa.gov/no2-pollution/basic-information-about-no2#What%20is%20NO2.

[91] European Environment Agency (2023). Harm to human health from air pollution in Europe: Burden of disease 2023. https://www.eea.europa.eu/publications/harm-to-human-health-from-air-pollution.

[92] de Bont J., Jaganathan S., Dahlquist M., Persson Å., Stafoggia M., Ljungman P. (2022). Ambient air pollution and cardiovascular diseases: An umbrella review of systematic reviews and meta-analyses. J. Intern. Med..

[93] Wang K., Yuan Y., Wang Q., Yang Z., Zhan Y., Wang Y., Wang F., Zhang Y. (2023). Incident risk and burden of cardiovascular diseases attributable to long-term NO_2_ exposure in Chinese adults. Environ. Int..

[94] Dedele A., Grazulevicienem R., Miskinyte A. (2017). Individual exposure to nitrogen dioxide and adverse pregnancy outcomes in Kaunas study. Int. J. Environ. Health Res..

[95] Ji X., Meng X., Liu C., Chen R., Ge Y., Kan L., Fu Q., Li W., Tse L.A., Kan H. (2019). Nitrogen dioxide air pollution and preterm birth in Shanghai, China. Environ. Res..

[96] US EPA, United States Environmental Protection Agency (2024). Sulfur Dioxide. Basics. https://www.epa.gov/so2-pollution/sulfur-dioxide-basics#what%20is%20so2.

[97] Orellano P., Reynoso J., Quaranta N. (2021). Short-term exposure to sulphur dioxide (SO_2_) and all-cause and respiratory mortality: A systematic review and meta-analysis. Environ. Int..

[98] Khalaf E.M., Mohammadi M.J., Sulistiyani S., Ramírez-Coronel A.A., Kiani F., Jalil A.T., Almulla A.F., Asban P., Farhadi M., Derikondi M. (2022). Effects of sulfur dioxide inhalation on human health: A review. Rev. Environ. Health..

[99] Wilkie A.A., Luben T.J., Rappazzo K., Foley K., Woods C.G., Serre M.L., Richardson D.B., Daniels J.L. (2024). Long-term ambient sulfur dioxide exposure during gestation and preterm birth in North Carolina, 2003–2015. Atmos. Environ. (1994)..

[100] Li L.L., Huang Y.H., Li J., Liu S., Chen Y.L., Jiang C.Z., Chen Z.J., Zhuang Y.Y. (2022). Maternal Exposure to Sulfur Dioxide and Risk of Omphalocele in Liaoning Province, China: A Population-Based Case-Control Study. Front. Public Health.

[101] European Environment Agency (2024). Sulphur Dioxide—Hourly Limit Value for the Protection of Human Health. https://www.eea.europa.eu/en/analysis/maps-and-charts/sulphur-dioxide-hourly-limit-value-for-the-protection-of-human-health-6.

[102] AQI, Air Quality Index https://www.aqi.in/dashboard/spain/basque-country/bilbao/europe/so2.

[103] Fussell J.C., Jauniaux E., Smith R.B., Burton G.J. (2024). Ambient air pollution and adverse birth outcomes: A review of underlying mechanisms. BJOG..

[104] Clemente D.B., Casas M., Vilahur N., Begiristain H., Bustamante M., Carsin A.E., Fernández M.F., Fierens F., Gyselaers W., Iñiguez C. (2016). Prenatal Ambient Air Pollution, Placental Mitochondrial DNA Content, and Birth Weight in the INMA (Spain) and ENVIRONAGE (Belgium) Birth Cohorts. Environ. Health Perspect..

[105] Movassaghi S., Jafari S., Falahati K., Ataei M., Sanati M.H., Jadali Z. (2020). Quantification of mitochondrial DNA damage and copy number in circulating blood of patients with systemic sclerosis by a qPCR-based assay. An. Bras. Dermatol..

[106] Gruzieva O., Xu C.J., Breton C.V., Annesi-Maesano I., Antó J.M., Auffray C., Ballereau S., Bellander T., Bousquet J., Bustamante M. (2017). Epigenome-Wide Meta-Analysis of Methylation in Children Related to Prenatal NO_2_ Air Pollution Exposure. Environ. Health Perspect..

[107] US EPA, United States Environmental Protection Agency (2024). Ground-Level Ozone Basics. https://www.epa.gov/ground-level-ozone-pollution/ground-level-ozone-basics.

[108] European Environment Agency (2024). Air Pollution. https://www.eea.europa.eu/en/topics/in-depth/air-pollution.

[109] Xue K., Zhang X. (2023). The rationale behind updates to ambient ozone guidelines and standards. Front. Public Health.

[110] Kim S.Y., Kim E., Kim W.J. (2020). Health Effects of Ozone on Respiratory Diseases. Tuberc. Respir. Dis..

[111] Rappazzo K.M., Nichols J.L., Rice R.B., Luben T.J. (2021). Ozone exposure during early pregnancy and preterm birth: A systematic review and meta-analysis. Environ. Res..

[112] Hao H., Yoo S.R., Strickland M.J., Darrow L.A., D’Souza R.R., Warren J.L., Moss S., Wang H., Zhang H., Chang H.H. (2023). Effects of air pollution on adverse birth outcomes and pregnancy complications in the U.S. state of Kansas (2000–2015). Sci. Rep..

[113] Li Y., Zhou C., Liu J., Mao D., Wang Z., Li Q., Wu Y., Zhang J., Zhang Q. (2024). Maternal Exposure to Ozone and the Risk of Birth Defects: A Time-Stratified Case-Crossover Study in Southwestern China. Toxics..

[114] US EPA, United States Environmental Protection Agency (2024). Basic Information About Carbon Monoxide (CO) Outdoor Air Pollution. https://www.epa.gov/co-pollution/basic-information-about-carbon-monoxide-co-outdoor-air-pollution#:~:text=The%20greatest%20sources%20of%20CO,can%20affect%20air%20quality%20indoors.

[115] Lee F.Y., Chen W.K., Lin C.L., Kao C.H. (2015). Carbon monoxide poisoning and subsequent cardiovascular disease risk: A nationwide population-based cohort study. Medicine.

[116] Statista Carbon Monoxide Emissions in the European Union (EU-27) from 1990 to 2022. https://www.statista.com/statistics/1171133/carbon-monoxide-emission-european-union-eu-28/#:~:text=Carbon%20monoxide%20(CO)%20emissions%20in,have%20fallen%20roughly%2070%20percent.

[117] Li H., Wu J., Wang A., Li X., Chen S., Wang T., Amsalu E., Gao Q., Luo Y., Yang X. (2018). Effects of ambient carbon monoxide on daily hospitalizations for cardiovascular disease: A time-stratified case-crossover study of 460,938 cases in Beijing, China from 2013 to 2017. Environ Health..

[118] Liu C., Yin P., Chen R., Meng X., Wang L., Niu Y., Lin Z., Liu Y., Liu J., Qi J. (2018). Ambient carbon monoxide and cardiovascular mortality: A nationwide time-series analysis in 272 cities in China. Lancet Planet. Health.

[119] Guo X., Song Q., Wang H., Li N., Su W., Liang M., Sun C., Ding X., Liang Q., Sun Y. (2022). Systematic review and meta-analysis of studies between short-term exposure to ambient carbon monoxide and non-accidental, cardiovascular, and respiratory mortality in China. Environ Sci Pollut Res Int..

[120] Ming X., Yang Y., Li Y., He Z., Tian X., Cheng J., Zhou W. (2024). Association between risk of preterm birth and long-term and short-term exposure to ambient carbon monoxide during pregnancy in Chongqing, China: A study from 2016–2020. BMC Public Health..

[121] Wang Y., Ruan Y., Wan X., Wang H., Guo J., Wei J., Ma S., He Y., Zou Z., Li J. (2024). Maternal exposure to ambient ozone and fetal congenital heart defects: A national multicenter study in China. J. Expo. Sci. Environ. Epidemiol..

[122] Wu T., Li Z., Wei Y. (2023). Advances in understanding mechanisms underlying mitochondrial structure and function damage by ozone. Sci. Total Environ..

[123] Wiegman C.H., Michaeloudes C., Haji G., Narang P., Clarke C.J., Russell K.E., Bao W., Pavlidis S., Barnes P.J., Kanerva J. (2015). Oxidative stress-induced mitochondrial dysfunction drives inflammation and airway smooth muscle remodeling in patients with chronic obstructive pulmonary disease. J. Allergy Clin. Immunol..

[124] Xu M., Wang L., Wang M., Wang H., Zhang H., Chen Y., Wang X., Gong J., Zhang J.J., Adcock I.M. (2019). Mitochondrial ROS and NLRP3 inflammasome in acute ozone-induced murine model of airway inflammation and bronchial hyperresponsiveness. Free Radic. Res..

[125] Rivas-Arancibia S., Hernández-Orozco E., Rodríguez-Martínez E., Valdés-Fuentes M., Cornejo-Trejo V., Pérez-Pacheco N., Dorado-Martínez C., Zequeida-Carmona D., Espinosa-Caleti I. (2022). Ozone Pollution, Oxidative Stress, Regulatory T Cells and Antioxidants. Antioxidants.

[126] Pelinsari S.M., Sarandy M.M., Vilela E.F., Novaes R.D., Schlamb J., Gonçalves R.V. (2024). Ozone Exposure Controls Oxidative Stress and the Inflammatory Process of Hepatocytes in Murine Models. Antioxidants.

[127] Alonso J.R., Cardellach F., López S., Casademont J., Miró O. (2003). Carbon monoxide specifically inhibits cytochrome c oxidase of human mitochondrial respiratory chain. Pharmacol. Toxicol..

[128] Hernansanz-Agustín P., Enríquez J.A. (2021). Generation of Reactive Oxygen Species by Mitochondria. Antioxidants.

[129] Coimbra-Costa D., Alva N., Duran M., Carbonell T., Rama R. (2017). Oxidative stress and apoptosis after acute respiratory hypoxia and reoxygenation in rat brain. Redox Biol..

[130] Angelova P.R., Myers I., Abramov A.Y. (2023). Carbon monoxide neurotoxicity is triggered by oxidative stress induced by ROS production from three distinct cellular sources. Redox Biol..

[131] Liu N., Xu H., Sun Q., Yu X., Chen W., Wei H., Jiang J., Xu Y., Lu W. (2021). The Role of Oxidative Stress in Hyperuricemia and Xanthine Oxidoreductase (XOR) Inhibitors. Oxid. Med. Cell Longev..

[132] Moghadam Z.M., Henneke P., Kolter J. (2021). From Flies to Men: ROS and the NADPH Oxidase in Phagocytes. Front. Cell Dev. Biol..

[133] González-Guevara E., Martínez-Lazcano J.C., Custodio V., Hernández-Cerón M., Rubio C., Paz C. (2014). Exposure to ozone induces a systemic inflammatory response: Possible source of the neurological alterations induced by this gas. Inhal. Toxicol..

[134] Sun L., Liu C., Xu X., Ying Z., Maiseyeu A., Wang A., Allen K., Lewandowski R.P., Bramble L.A., Morishita M. (2013). Ambient fine particulate matter and ozone exposures induce inflammation in epicardial and perirenal adipose tissues in rats fed a high fructose diet. Part. Fibre Toxicol..

[135] Iacobellis G., Bianco A.C. (2011). Epicardial adipose tissue: Emerging physiological, pathophysiological and clinical features. Trends Endocrinol. Metab..

[136] Deshmane S.L., Kremlev S., Amini S., Sawaya B.E. (2009). Monocyte chemoattractant protein-1 (MCP-1): An overview. J. Interferon Cytokine Res..

[137] Ramos-Lobo A.M., Donato J. (2017). The role of leptin in health and disease. Temperature.

[138] Phillips S.A., Kung J.T. (2010). Mechanisms of adiponectin regulation and use as a pharmacological target. Curr. Opin. Pharmacol..

[139] Tian L., Li N., Li K., Tan Y., Han J., Lin B., Lai W., Liu H., Shi Y., Xi Z. (2022). Ambient ozone exposure induces ROS related-mitophagy and pyroptosis via NLRP3 inflammasome activation in rat lung cells. Ecotoxicol. Environ. Saf..

[140] Lu L., Zhang Y., Tan X., Merkher Y., Leonov S., Zhu L., Deng Y., Zhang H., Zhu D., Tan Y. (2022). Emerging mechanisms of pyroptosis and its therapeutic strategy in cancer. Cell Death Discov..

[141] Miller C.N., Dye J.A., Schladweiler M.C., Richards J.H., Ledbetter A.D., Stewart E.J., Kodavanti U.P. (2018). Acute inhalation of ozone induces DNA methylation of apelin in lungs of Long-Evans rats. Inhal. Toxicol..

[142] Wu Y., Wang X., Zhou X., Cheng B., Li G., Bai B. (2017). Temporal Expression of Apelin/Apelin Receptor in Ischemic Stroke and its Therapeutic Potential. Front. Mol. Neurosci..

[143] O’Carroll A.M., Lolait S.J., Harris L.E., Pope G.R. (2013). The apelin receptor APJ: Journey from an orphan to a multifaceted regulator of homeostasis. J. Endocrinol..

[144] Xia Y., Niu Y., Cai J., Lin Z., Liu C., Li H., Chen C., Song W., Zhao Z., Chen R. (2018). Effects of Personal Short-Term Exposure to Ambient Ozone on Blood Pressure and Vascular Endothelial Function: A Mechanistic Study Based on DNA Methylation and Metabolomics. Environ. Sci. Technol..

[145] Su C., Xue J., Ye C., Chen A. (2021). Role of the central renin-angiotensin system in hypertension (Review). Int. J. Mol. Med..

[146] Banecki K.M.R.M., Dora K.A. (2023). Endothelin-1 in Health and Disease. Int. J. Mol. Sci..

[147] Kalia P., Ansari M.A. (2020). IOT based air quality and particulate matter concentration monitoring system. Mater. Today: Proc..

[148] Kim H., Tae S., Zheng P., Kang G., Lee H. (2021). Development of IoT-based particulate matter monitoring system for construction sites. Int. J. Environ. Res. Public Health.

[149] Jo J.H., Jo B., Kim J.H., Choi I. (2020). Implementation of iot-based air quality monitoring system for investigating particulate matter (Pm10) in subway tunnels. Int. J. Environ. Res. Public Health.

[150] Divan M.J., Sanchez-Reynoso M.L., Panebianco J.E., Mendez M.J. (2021). IoT-based approaches for monitoring the particulate matter and its impact on health. IEEE Internet Things J..

[151] Naik U.U., Salgaokar S.R., Jambhale S. (2023). IOT based air pollution monitoring system. Int. J. Sci. Res. Eng. Trends.

[152] Mabrouki J., Azrour M., Dhiba D., Farhaoui Y., El Hajjaji S. (2021). IoT-based data logger for weather monitoring using arduino-based wireless sensor networks with remote graphical application and alerts. Big Data Min. Anal..

[153] De Medeiros H.P.L., Girão G. (2020). An IoT-based air quality monitoring platform. Proceedings of the 2020 IEEE International Smart Cities Conference (ISC2).

[154] Almalawi A., Alsolami F., Khan A.I., Alkhathlan A., Fahad A., Irshad K., Qaiyum S., Alfakeeh A.S. (2022). An IoT based system for magnify air pollution monitoring and prognosis using hybrid artificial intelligence technique. Environ. Res..

[155] Kumar S., Jasuja A. (2017). Air quality monitoring system based on IoT using Raspberry Pi. Proceedings of the 2017 International Conference on Computing, Communication and Automation (ICCCA).

[156] Ghadge A., Juvekar A., Wakode M., Kale G. (2023). Carbon Monoxide Concentration Monitoring System for Automating Air Filters. Proceedings of the 2023 International Conference on Emerging Smart Computing and Informatics (ESCI).

